# In Situ Fe-Doped Hierarchically Porous Carbon Derived from *Astragalus* and *Licorice* Residues: Pore Engineering and Thermal Conductivity Enhancement for Methanol Adsorption Refrigeration

**DOI:** 10.3390/ma19153281

**Published:** 2026-08-03

**Authors:** Xujin Gong, Xiang Zou, Wenlan Li, Yi Zhao

**Affiliations:** 1 Postdoctoral Programme of Materia Medica Institute, Harbin University of Commerce, Harbin 150076, China; kimkung@126.com; 2 School of Energy and Civil Engineering, Harbin University of Commerce, Harbin 150028, China; zhaoyi9007020@163.com

**Keywords:** traditional Chinese medicine residues, hierarchically porous carbon, in situ Fe doping, pore regulation, adsorption refrigeration, low-grade waste heat recovery

## Abstract

Approximately 40–70 million tons of traditional Chinese medicine (TCM) residues are produced annually in China, which poses severe environmental challenges. Meanwhile, adsorption refrigeration driven by low-grade heat is limited by the lack of high-performance, low-cost adsorbents. Herein, in situ Fe-doped hierarchically porous carbon (denoted as ZYC-ZFe) was fabricated using residues of *Astragalus membranaceus* (55 wt%) and *Glycyrrhiza uralensis* (45 wt%). The resultant material exhibited a Brunauer–Emmett–Teller specific surface area (*S*_BET_) of 1244.69 m^2^/g, a total pore volume (*V*_T_) of 1.123 cm^3^/g, a thermal conductivity of 4.517 W/(m·K), and a maximum Langmuir methanol adsorption capacity of 713.06 mg/g. A desorption efficiency of 97.6% was realized within 30 min at 90 °C, and only 3.2% reduction in adsorption capacity was observed after 100 consecutive adsorption–desorption cycles. In scaled-up CFD simulations using adsorbent beds of different sizes, the adsorption refrigeration bed packed with ZYC-ZFe shortened the core temperature rise time. Within a 30 min working cycle, ZYC-ZFe delivered a specific cooling power of 1534.6 kJ/(kg·h) and a coefficient of performance (*COP*) of 0.52 at 90 °C. This study offers a feasible technical strategy for the high-value reutilization of TCM residues and facilitates the practical application of adsorption refrigeration driven by low-grade waste heat.

## 1. Introduction

Since the Industrial Revolution, the conflict between economic growth and climate degradation has become increasingly prominent, driving the pursuit of sustainable technological innovation and industrial green transformation [1]. As a proactive response, China has built a distinctive institutional system balancing social progress and climate governance, adopting targeted measures to deliver its Dual Carbon goals (carbon peaking by 2030, carbon neutrality by 2060) [2]. Nonetheless, many sectors still face prominent environmental constraints in the process of green transition. As a strategic pillar of modern biomedicine, the traditional Chinese medicine (TCM) industry is pursuing modernization, intelligent upgrading, and green restructuring, yet annual discharge of 40 million tons of TCM residues has become a severe environmental burden and technical bottleneck [3]. The annual output is projected to reach 60–70 million tons with industrial development.

Current disposal of TCM residues primarily relies on landfilling, open stockpiling, and incineration, which bring a range of ecological problems, such as land occupancy, groundwater contamination, and toxic flue gas release [4,5]. Despite developed biomass energy conversion technologies, most suffer from low resource and economic efficiency, leaving TCM residue potential underutilized [6]. Notably, TCM residues are hazardous industrial solid wastes, yet they are also abundant, underutilized renewable lignocellulosic raw materials [3,7], so efficient technologies for their harmless treatment and high-value reutilization are urgently needed. Fabricating high-performance functional carbon from waste biomass has become a mainstream direction in biomass valorization research [8,9,10]. Hierarchically porous biocarbons synthesized from TCM-derived lignocellulose exhibit promising application prospects in energy storage, environmental remediation, and heterogeneous catalysis.

Meanwhile, adsorption refrigeration is a competitive low-carbon alternative to conventional vapor-compression refrigeration, as it features ultra-low power consumption (merely one-third of that of conventional compression refrigeration), ozone-safe working media, and low operational noise, aligning with global carbon reduction targets [11]. Distinct from vapor-compression refrigeration, which demands massive high-grade electricity, adsorption refrigeration employs readily accessible low-grade thermal energy, such as solar and industrial waste heat for cooling via reversible adsorption–desorption of refrigerants on porous adsorbents [12,13].

Porous carbons represent the most promising adsorbents for adsorption refrigeration, coupling high-value conversion of biomass with low-carbon cooling [14,15,16]. However, commercial activated carbon has limited adsorption capacity, slow kinetics, and low thermal conductivity, drastically reducing refrigeration system efficiency [17,18]. To mitigate the inherent limitations of carbon adsorbents, metal doping has emerged as an effective modification route. Our prior work fabricated in situ Fe-doped magnetic carbon (IM-WNC) using municipal sludge as precursors [19]. The optimized samples exhibit favorable pore structure, thermal conductivity, and methanol adsorption capacity. These findings verify that in situ Fe doping represents a promising approach to boost the adsorption refrigeration performance of activated carbons and biocarbons [20].

Nevertheless, precursors adopted in the above research are restricted to municipal sludge, and the combination of Fe doping and TCM residue-derived biocarbons remains unexplored. Against municipal sludge, TCM residues stand out as attractive precursors for adsorbent biocarbons [21]. The resulting carbon material possesses a rigid skeleton enabling stable cyclic adsorption [22,23]. Meanwhile, the feedstock requires minimal pretreatment and enjoys sufficient, centralized supply. Moreover, few efforts have been devoted to developing TCM residue-derived carbon adsorbents tailored for low-grade heat-driven methanol adsorption refrigeration, creating a distinct research gap bridging the high-value utilization of herbal waste and the innovation of high-performance refrigeration adsorbents [24,25].

To fill the research gaps, this work adopts precursor compounding, in situ Fe templating, and two-stage activation, and completes four core research tasks: (1) screen and optimize mixed TCM residue precursors; (2) establish an integrated carbon fabrication process and reveal parameter-controlled pore evolution via response surface methodology (RSM); (3) quantitatively correlate pore architecture, surface chemistry, and methanol uptake via multi-scale characterizations; (4) assess long-cycle refrigeration performance and benchmark against commercial adsorbents.

Beyond the above research tasks, this study targets two core scientific challenges rarely solved synchronously in existing biomass carbon research: balancing saturated adsorption capacity and rapid mass transfer through matched micropore–mesopore hierarchical construction; and elucidating the multifunctional regulatory roles of in situ generated Fe_3_O_4_ nanoparticles in pore shaping, surface modification, and thermal conductivity enhancement for low-grade waste heat refrigeration.

This interdisciplinary work bears ecological, academic, and industrial value: ecologically, it achieves clean high-value recycling of herbal wastes and eliminates sludge pyrolysis secondary pollution; academically, it creates a new linkage between lignocellulosic waste valorization and low-grade adsorption refrigeration to break sludge carbon’s inherent limitations; industrially, it provides stable high-performance adsorbents for waste heat recovery and advances both the green transformation of the TCM sector and China’s Dual Carbon strategy.

## 2. Materials and Methods

### 2.1. Raw Materials

#### 2.1.1. TCM Residues

Four types of herbal extraction residues were collected from a standardized TCM processing factory in Harbin, China: *Astragalus membranaceus* (shortened as AM), *Glycyrrhiza uralensis* (GU), *Pueraria lobata* (PL), and *Angelica sinensis* (AS). These residues were selected based on high annual yield and high lignocellulose content. Combined, the four residues make up over 22% of China’s total annual solid waste output from TCM manufacturing. The substantial and stable annual output ensures a reliable, scalable feedstock supply for large-scale resource valorization and industrial applications. All residues were stored in a dry and dark environment at 4 °C, and pretreated within 7 days to prevent microbial degradation. Table 1 lists the detailed chemical compositions of the four TCM residues on a dry weight basis. All component tests followed the standard biomass analysis protocol NREL/TP-510-42618 [26].

#### 2.1.2. Chemical Reagents

All reagents were of analytical grade and used without further purification. Zinc chloride (ZnCl_2_, ≥98.0 wt%), ferrous sulfate heptahydrate (FeSO_4_·7H_2_O, ≥99.0 wt%), and phosphoric acid (H_3_PO_4_, 85 wt%) were purchased from Sinopharm Chemical Reagent Co., Ltd. (Shanghai, China). Anhydrous methanol (CH_3_OH, ≥99.5 wt%) served as the adsorbate. Sodium hydroxide (NaOH), sodium bicarbonate (NaHCO_3_), and hydrochloric acid (HCl) were used for Boehm titration (Sinopharm Chemical Reagent Co., Ltd., Shanghai, China). Deionized water was used throughout the experiments. CH_3_OH, NaOH and HCl were also purchased from Sinopharm Chemical Reagent Co., Ltd. (Shanghai, China).

### 2.2. Synthesis of Fe-Doped Hierarchically Porous Carbon

#### 2.2.1. Screening and Blending of Precursor Materials

**Figure 1:**
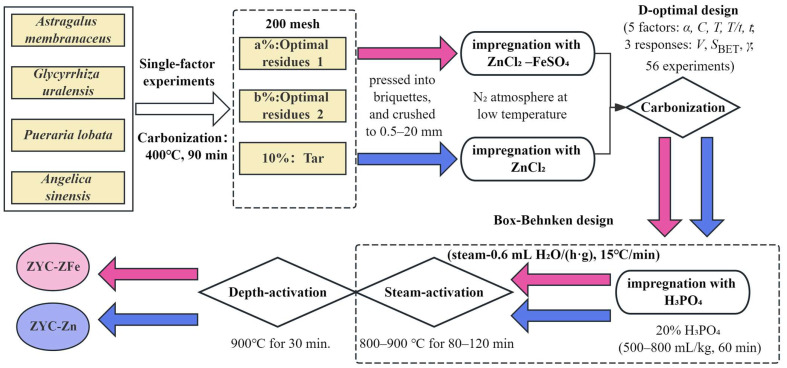
Schematic diagram of the preparation process for TCM residue-derived biocarbons. Key experimental details: four TCM residues undergo single-factor pre-carbonization (400 °C, 90 min) to screen the optimal blend with 10% tar binder; pulverized 200-mesh powder is split into ZnCl_2_–FeSO_4_ co-impregnation or pure ZnCl_2_ impregnation under N_2_ protection; carbonization parameters are optimized via D-optimal design (5 factors, 56 tests); and subsequently, two-stage steam activation (0.6 mL/(h·g) steam, 15 °C/min ramp) with H_3_PO_4_ impregnation is tuned by Box–Behnken design to produce hierarchical porous adsorbents for methanol adsorption refrigeration.

The single-factor method was used to explore the effect of blending ratios of TCM residues on the performance of carbon materials prepared via low-temperature carbonization at 400 °C for 90 min. Six blending mass ratios were designed for AM and GU residues: 100:0, 75:25, 55:45, 45:55, 25:75, and 0:100. We added 10 wt% tar as a binder during granulation. The pore volume (*V_T_*) and specific surface area (*S*_BET_) were comprehensively analyzed to determine the optimal combination of two TCM residues and their matching mass proportions (*a*% and *b*% in Figure 1).

#### 2.2.2. Precursor Pretreatment

Each selected TCM residue was rinsed three times with deionized water to remove soluble impurities, and then dried in a blast oven at 80 °C until a constant weight was achieved. Dried residues were pulverized and sieved through a 200-mesh screen (aperture size: 0.074 mm). The screened powder was tightly sealed for storage to prevent moisture absorption and external contamination. Based on the optimal ratio (55% AM residue and 45% GU residue), 10 wt% tar was incorporated as a binder.

#### 2.2.3. Combined Impregnation and Carbonization

The granular carbon precursors were impregnated with a mixed ZnCl_2_–FeSO_4_ solution. The molar ratio of FeSO_4_·7H_2_O to ZnCl_2_ was fixed at 1:1. After adjusting the impregnation ratio to the preset value and ensuring sufficient mixing for complete impregnation, solid–liquid separation was carried out to collect the impregnated precursors. The detailed parameters of the combined impregnation process are presented in the subsequent D-optimal experimental design. Preliminary tests prove that carrying out impregnation in a low-temperature aqueous solution under nitrogen protection can effectively suppress the oxidation of Fe(II) ions. This treatment improves iron salt loading inside precursor pores and raises the iron oxide content of final carbonized products. Consequently, the entire impregnation process was performed under a nitrogen atmosphere to guarantee the stability of the impregnant and the quality of the impregnated precursors.

For the carbonization process, the impregnated carbon precursor samples were placed in a quartz boat, which was then pushed into the constant-temperature zone of a tube furnace (Anhui Kemi Instrument Co., Ltd., Hefei, China). Before heating, high-purity nitrogen (≥99.99% purity) was pumped into the tube furnace to fully purge internal air, preventing oxidative decomposition of precursors during carbonization. Subsequently, the system was heated to the preset carbonization temperature at a controlled rate and maintained at this temperature for a specified holding duration. After the carbonization process was completed, heating was terminated, and the samples were naturally cooled to room temperature under a continuous nitrogen flow to obtain the final carbonized materials.

A D-optimal experimental design was adopted in this stage to systematically investigate the multi-factor and multi-level effects of key process parameters on the properties of the carbonized materials. Based on the results of prior single-factor preliminary experiments, five critical independent variables and their corresponding level ranges were selected, as detailed in Table 2.

With *V_T_*, *S*_BET_, and *γ* as the response indicators, a total of 56 experiments were designed, including 3 replicated validation tests to verify the reliability of the experimental results. The D-optimal design was specifically chosen for the carbonization stage because the asymmetric ranges of the impregnation parameters identified in the preliminary experiments could not be accommodated by traditional orthogonal designs, which require equal-interval grouping of parameter levels.

#### 2.2.4. Phosphoric Acid Impregnation and Steam Activation

After natural cooling to room temperature under continuous nitrogen flow, carbonized samples were soaked in 20 wt% H_3_PO_4_ solution for a fixed 60 min. The phosphoric acid impregnation ratio (defined as *β*) varied from 500 to 800 mL/kg to guarantee full penetration of the activating agent into the carbon matrix. After impregnation, solid–liquid separation was performed to collect the impregnated carbonized materials, which were subsequently dried at a low temperature to remove residual moisture without altering the pore structure formed during carbonization. The dried impregnated carbonized materials were transferred to an activation furnace for steam activation. The activation process was conducted with a steam flow rate of 0.6 mL/(h·g) and a controlled heating rate of 15 °C/min. The activation process consisted of two sequential stages: in the first stage, the temperature was maintained at 800 °C for a holding time of 80–120 min to initiate pore formation and structural rearrangement; in the second stage (depth activation), the temperature was further elevated to 900 °C and held for 30 min to enhance pore development and optimize the porous structure. Through this two-stage activation process, composite carbon materials derived from TCM residues via combined impregnation and activation were successfully obtained.

To systematically optimize the activation process parameters and their interactions, a three-level Box–Behnken design (BBD) was employed for the three-factor system in the activation stage, which included the impregnation ratio, activation temperature, and activation time. A total of 17 experimental runs were designed. The independent variables in the BBD were defined as follows: impregnation ratio (*β*), activation temperature (*T*_h_), and activation time (*t*_h_). *V_T_* and *S*_BET_ were selected as the key response indicators to evaluate the porous structure and performance of the activated carbon materials. The TCM residue-based composite carbon material prepared under the optimal process parameters jointly determined by the D-optimal design (for carbonization) and the Box–Behnken design (for activation) was designated as ZYC-ZFe. For comparison, a control carbon material (ZYC-Zn) was also prepared by impregnation in a single ZnCl_2_ solution.

### 2.3. Characterization Methods

Nitrogen adsorption–desorption isotherms were measured at 77 K using an ASAP 2020 surface area and porosity analyzer (Micromeritics, Norcross, GA, USA). The Brunauer–Emmett–Teller (BET) specific surface area, total pore volume, and pore size distribution of the carbon materials were calculated via the BET model, the t-Plot method, and the Barrett–Joyner–Halenda (BJH) model, respectively. Thermal conductivity was tested via a DRXL-I steady-state plate thermal conductivity analyzer (Xiangtan Instrument Co., Ltd., Xiangtan, China). Approximately 30 g of granular carbon samples was filled into an annular fixture at a packing density of 520 ± 10 kg/m^3^ to simulate practical adsorbent bed conditions. A steady temperature gradient of ca. 10 °C was applied over the sample, with the hot plate held at 35 °C and the cold plate at 25 ± 0.5 °C. Once thermal equilibrium was attained (temperature drift < 0.1 °C within 10 min), 15 successive readings were collected at 1 min intervals and averaged. Five replicate tests were conducted per sample (*n* = 5), and all results are presented as the mean ± standard deviation. The apparatus was calibrated with certified Pyrex glass (λ = 1.14 W/(m·K)), yielding measurement deviations below ± 3%. The burn-off rate was calculated based on the mass loss of samples measured with an ME204E analytical balance (Mettler Toledo, Switzerland). Morphological observation was performed using an SU8010 scanning electron microscope (SEM, Hitachi, Tokyo, Japan); surface chemical composition analysis was carried out via an ESCALAB 250Xi X-ray photoelectron spectrometer (XPS, Thermo Scientific, Madison, MA, USA); surface functional group detection was conducted using an iS50 Fourier transform infrared spectrometer (FTIR, Nicolet, Madison, WI, USA); and elemental composition analysis was performed with a Vario EL III elemental analyzer (Elementar, Langenselbold, Germany).

### 2.4. Methanol Adsorption–Desorption Measurements

**Figure 2:**
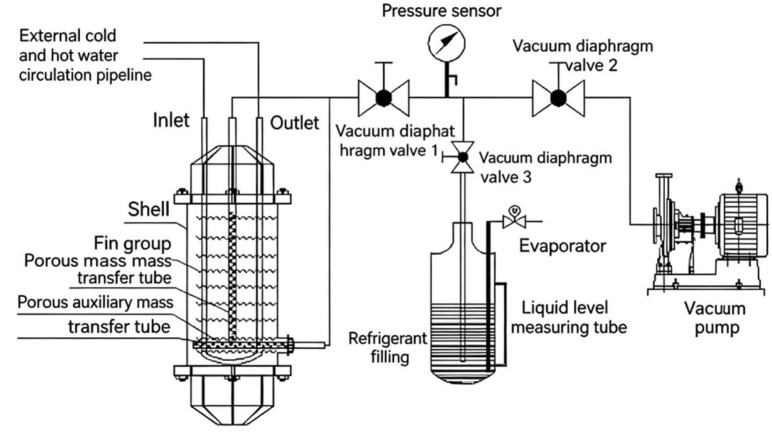
Schematic of the experimental setup for methanol adsorption–desorption tests, which enables real-time quantification of methanol adsorption capacity and long-cycle adsorption–desorption performance. It consists of hot and cold water circulation loops, a finned adsorption bed, an evaporator with a high-precision liquid level meter, vacuum diaphragm valves, and a vacuum pump.

A custom-built liquid-level adsorption–desorption test apparatus reported in our previous work was employed to measure the adsorption rate curves of carbon–methanol working pairs [19,27,28]. As shown in Figure 2, it is equipped with independent heating and cooling circulation loops, as well as a high-precision liquid level monitoring system, which enables accurate quantification of methanol adsorption/desorption capacity and long-term cycle performance of the carbon materials. The adsorption bed chiller was designed with a cylindrical structure of *φ*150 mm × 530 mm, with a carbon bed height of 280 mm. It was integrated with heat-conducting fins, U-shaped heat transfer tubes, and primary/secondary porous refrigerant mass transfer tubes to enhance heat and mass transfer efficiency. The evaporator had an effective volume of 1.5 L, which was paired with a high-precision liquid-level meter (measurement range: 0–1500 mL) for real-time monitoring of methanol volume changes. Based on the findings of prior relevant research, the total duration of a single methanol adsorption/desorption cycle was set to 30 min, with the heating medium temperature for desorption maintained at a constant 100 °C and the cooling water temperature for adsorption controlled at 23 ± 2 °C to ensure test reproducibility. The system pressure was maintained at 10 kPa during adsorption and 100 kPa during desorption. The packing density of the adsorbent bed was optimized to 520 kg/m^3^, which balances the heat transfer efficiency and mass transfer resistance of the bed.

The detailed procedure of the methanol adsorption/desorption cycle test is described as follows:

(1) Material Loading and Vacuum Extraction: The degassed carbon material (MAC = 1.35 kg, degassed at 200 °C for 12 h) was loaded into the adsorption chiller according to the test batch, while liquid methanol refrigerant (0.8 kg) was loaded into the evaporator. The heating medium circulation system was activated, and the chiller bed was heated via the U-shaped heat transfer tube. After pressure stabilization, vacuum diaphragm valves 1 and 2 were opened, and the system was evacuated using an OLF750AF two-stage vacuum pump. Once the system pressure reached the preset vacuum value, valves 1 and 2, the vacuum pump, and the heating medium circulation system were shut down sequentially. Vacuum diaphragm valve 3 was then opened, and the system pressure was continuously monitored for 24 h. If the pressure remained stable within the allowable error range and met the design requirements, the initial methanol volume (*V*_0_, mL) in the evaporator was accurately recorded.
(2) Adsorption Refrigeration Process: The adsorption chiller bed was cooled by activating the cooling water circulation loop to the preset temperature (23 ± 2 °C), and then vacuum diaphragm valves 1 and 3 were opened. As the methanol vapor was continuously adsorbed by the carbon material in the adsorption bed, the liquid level in the evaporator gradually decreased. After 30 min of stable adsorption, the liquid level volume (*V*_1_, mL) in the evaporator was recorded. The methanol adsorption capacity (*Q*_ad_, mg/g) was calculated using the following formula:

(1) $$Q_{\mathrm{ad}}=\frac{\rho_{\mathrm{methanol}}\left(V_{0}-V_{1}\right)}{M_{AC}}$$

(3) Heating and Desorption Process: Vacuum diaphragm valves 1 and 3 were closed, and then the system was switched to the heating medium circulation loop to heat the adsorption bed to 100 °C. Upon reaching the preset condensation pressure, valves 1 and 3 were reopened, allowing the desorbed methanol vapor to flow into the evaporator and condense into a liquid phase continuously. After 30 min of stable desorption, the methanol liquid level volume (*V*_2_, mL) in the evaporator was recorded. The methanol desorption capacity (*Q*_de_, mg/g) was calculated using the following formula:

(2) $$Q_{\mathrm{ad}}=\frac{\rho_{\mathrm{methanol}}\left(V_{0}-V_{1}\right)}{M_{AC}}$$

To ensure the reliability and reproducibility of the test results, the adsorption and desorption capacities were determined by averaging the data obtained from three consecutive stable test cycles.

### 2.5. Adsorption Models and Calculation Methods

#### 2.5.1. Adsorption Rate Calculation (Sokoda–Suzuki Model)

The methanol adsorption and desorption rates were calculated using the Sokoda–Suzuki kinetic model [27,29], which is widely recognized for describing mass transfer processes in porous adsorbent systems. The model equations are expressed as follows:

(3) $$Q(t)=Q^{*}-\left(Q^{*}-Q_{\mathrm{ini}}\right)\;\exp\left(-kt\right)$$

(4) $$k=\frac{15D_{S0}}{R_{p}^{2}}\exp\frac{E_{a}}{RT}$$

wherein *Q* represents the methanol adsorption/desorption amount at time *t* (mg/g); *t* denotes the reaction time (s); Q* is the equilibrium adsorption/desorption amount (mg/g); *k* is the adsorption rate constant (s^−1^); *D*_So_ stands for the surface diffusion coefficient (m^2^/s); *E*_a_ is the activation energy for surface diffusion (J/mol); *R*_p_ is the average particle diameter of the carbon material (m); and *T* is the absolute temperature (K). This model effectively describes the kinetic behavior of methanol adsorption on porous carbon materials, comprehensively accounting for surface diffusion effects and thermal activation barriers that influence mass transfer efficiency.

#### 2.5.2. Adsorption Isotherm Fitting

The adsorption equilibrium data obtained from the cycle tests were fitted using two classical adsorption models, the Langmuir model and the Dubinin–Astakhov (D–A) [30] model, to clarify the adsorption mechanism of methanol on the TCM residue-derived carbon materials. The Langmuir model is derived from kinetic theory and assumes monolayer adsorption on a homogeneous adsorbent surface, making it suitable for chemisorption-dominated adsorption processes. In contrast, the D–A model is based on Polanyi’s adsorption potential theory and is particularly applicable to microporous adsorbents (e.g., activated carbon) with heterogeneous surfaces, which is consistent with the structural characteristics of the prepared carbon materials.

(5) $$Q_{L}=\frac{k_{L}\times {Q_{L}}^{*}\times (\frac{p}{p_{0}})}{1+k_{L}(\frac{p}{p_{0}})}$$

(6) $$Q_{D-A}={Q_{D-A}}^{*}\exp[-{\left(\frac{\varepsilon }{E}\right)}^{n}]$$

wherein *Q*_L_ is the methanol adsorption/desorption amount (mg/g); *k*_L_ is the adsorption constant related to the binding affinity between methanol molecules and the carbon surface; *Q*_L_* represents the maximum monolayer adsorption capacity (mg/g); *p* is the actual gas pressure (Pa); *p*_0_ is the saturation vapor pressure of methanol at the test temperature (Pa); *Q*_D-A_ is the methanol adsorption/desorption amount (mg/g); *Q*_D-A_^*^ is the maximum adsorption/desorption amount (mg/g); *ε* is the adsorption potential (*ε = RT*ln(*p*_0_/*p*); *E* is the characteristic adsorption energy (J/mol); and *n* is an empirical parameter, which is closely related to the heterogeneity of the adsorbent surface.

#### 2.5.3. Thermodynamic Equations

The Gibbs free energy change (*Δ*G) was determined from the adsorption equilibrium constant *K*_L_ obtained from the Langmuir isotherm model (Equation (5)):

(7) $$\Delta G=-RT\ln K_{L}$$

wherein *R* is the universal gas constant (8.314 J/(mol·K)), *T* is the absolute temperature (K), and *K*_L_ is the Langmuir adsorption constant.

The enthalpy change (Δ*H*) and entropy change (Δ*S*) were derived from the van’t Hoff equation. The values of ΔH and ΔS were determined from the slope (−ΔH/R) and intercept (Δ*S*/*R*) of the linear regression of ln K_L_.

(8) $$\ln K_{L}=-\frac{\Delta H}{RT}+\frac{\Delta S}{R}$$

The activation energies for adsorption (*E*_a_) and desorption (*E*_d_) were calculated from the temperature dependence of the surface diffusion coefficient *D*_S0_ using the Arrhenius equation:

(9) $$D_{s0}=D_{0}\;\exp\;(-\frac{E_{a}}{RT})$$

wherein *D*_0_ is the pre-exponential factor (m^2^/s). For desorption, *E*a is replaced by *E*_d_ accordingly.

#### 2.5.4. Cooling Capacity and Power Calculation

The cooling performance of the adsorption refrigeration system under different operating conditions was evaluated by calculating the cooling capacity and cooling power using the experimental data obtained from the methanol adsorption–desorption cycles. The calculation formulas are as follows [31]:

(10) $$Q_{ref}=\frac{V\times \Delta H_{ev}}{W_{ad}}$$

(11) $$N_{ref}=\frac{Q_{ref}}{t_{ad}}$$

wherein *Q*_ref_ is the cooling capacity per unit mass of adsorbent (kJ/kg); *V* is the desorbed refrigerant volume per kilogram of carbon material (L/kg); *ΔH*_ev_ is the latent heat of the vaporization of methanol at the evaporation temperature (J/mL); *W*_ad_ is the mass of the carbon adsorbent (kg); *N*_ref_ represents the cooling power (kJ/(kg·h)); and *t*_ad_ is the total duration of a single adsorption/desorption cycle (min).

### 2.6. Computational Fluid Dynamics (CFD) Simulation of Scaled-Up Adsorbent Beds

To evaluate the dynamic heat transfer characteristics of carbon adsorbents at larger scales beyond the laboratory setup (φ150 mm × 530 mm), CFD simulations were performed for three cylindrical adsorbent beds with inner diameters of 100, 200, and 400 mm (length: 1000 mm), packed with granular carbon at a density of 520 kg/m^3^. The geometry and heat transfer tube configuration were consistent with the laboratory-scale chiller described in Section 2.4. A 2D axisymmetric model was constructed in ANSYS Fluent 2024R1 (Version 24.1.0). The transient heat transfer was governed by the energy conservation equation with a local thermal non-equilibrium model, and the adsorption/desorption heat source was incorporated via the linear driving force (LDF) model using kinetic parameters (*D*_S0_, *E*_a_) obtained from the experimental Sokoda–Suzuki fitting. The boundary conditions included a constant heating water temperature of 370 K at the tube wall (convective heat transfer coefficient: 500 W/(m^2^·K)) and adiabatic outer walls. The initial bed temperature was set to 300 K. The CFD model was validated against experimental temperature data from the *φ*150 mm bed (deviation < 5%), and a grid independence study confirmed mesh sizes of 151,601, 268,856, and 393,427 cells for the 100, 200, and 400 mm tubes, respectively, all with mesh quality > 0.95.

## 3. Results and Discussion

### 3.1. Precursor Screening and Carbonization-Induced Pore Evolution

#### 3.1.1. Binary Precursor Optimization and Lignocellulosic Synergy

The carbonization stage defines the primary carbon skeleton and initial pore architecture, which fundamentally constrains subsequent activation efficiency and final textural performance [32]. Single-factor experiments were conducted to explore the effect of blending ratios on the porous properties of carbonized products. The total lignocellulose content (TLC), *V*_T_, and *S*_BET_ of all single-component and binary blended samples are summarized in Table 3.

As shown in Table 3, the single AM and GU residues deliver total pore volumes of only 0.404 cm^3^/g and 0.472 cm^3^/g, with corresponding specific surface areas of 524.26 m^2^/g and 572.98 m^2^/g, respectively. PL and AS present even inferior textural characteristics, with the lowest pore volume (0.267 cm^3^/g) and specific surface area (386.27 m^2^/g) observed for AS. In contrast, binary co-blending of AM and GU produces an obvious synergistic effect on pore formation and structural development. Among all tested mixing ratios, the sample with 55 wt% AM and 45 wt% GU achieves the optimal porous architecture. Its total pore volume reaches 0.461 cm^3^/g and the *S*_BET_ is 562.17 m^2^/g, both outperforming most single-component resins and other AM/GU binary combinations.

**Figure 3:**
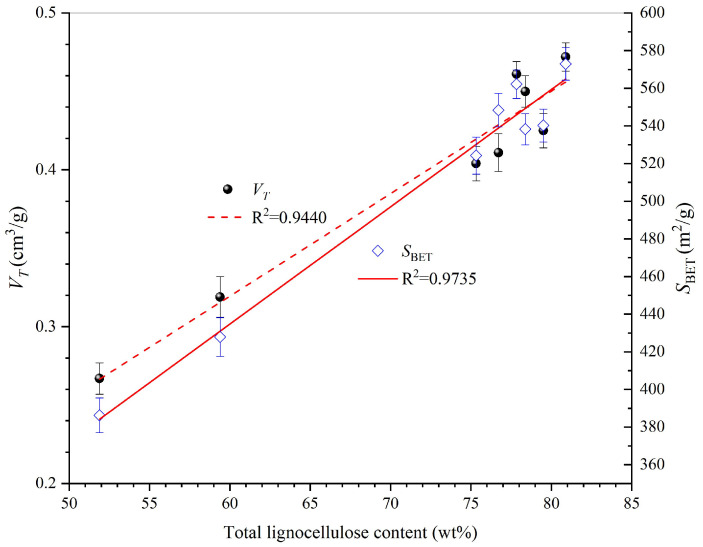
Correlation of lignocellulose content with pore characteristics. Linear fitting curves show the positive correlation between total lignocellulose content (TLC, wt%) and two critical pore indicators of carbonized TCM residue precursors: total pore volume *V*_T_ (*R*^2^ = 0.9440) and BET specific surface area *S*_BET_ (*R*^2^ = 0.9735).

The differences in porous performance are closely associated with the TLC of raw materials, which is defined as the sum of cellulose, hemicellulose, and lignin. The optimal 55 wt% AM + 45 wt% GU blend has a TLC of 77.83 wt%, which is remarkably higher than those of PL (59.39 wt%) and AS (51.89 wt%). To further quantify the internal relationship between lignocellulose composition and pore characteristics, linear regression analysis was performed, and the results are presented in Figure 3. A strong positive correlation was observed between the TLC and the porous performance of carbonized products. The coefficients of determination (*R*^2^) are 0.9440 for total pore volume and 0.9735 for specific surface area, demonstrating that lignocellulosic components are the dominant factor governing pore evolution during carbonization. The low lignocellulose content of PL and AS leads to incomplete thermal decomposition, insufficient release of volatile components, and discontinuous pore networks, ultimately resulting in unsatisfactory porous structures.

The excellent performance of the AM–GU blend originates from the complementary lignocellulosic compositions and distinct thermochemical behaviors of the two residues. AM contains a high cellulose content of 43.82 wt%. Cellulose undergoes gradual thermal decomposition at 240–350 °C, releasing volatile fragments while maintaining a rigid carbon framework [33,34]. This stable skeleton well preserves the intrinsic microporous structure and endows the carbonized product with a high specific surface area. Differently, GU is rich in hemicellulose (25.21 wt%) and lignin (20.12 wt%). Hemicellulose decomposes rapidly at 180–280 °C, and the escape of abundant volatile organics carves interconnected mesoporous channels within the carbon matrix. Lignin decomposes at 280–450 °C, which promotes cross-linking of the carbon skeleton, enhances structural integrity, and effectively suppresses premature collapse of the pore network [33].

#### 3.1.2. Carbonization Parameter Optimization via D-Optimal Design and Mechanistic Interpretation

**Figure 4:**
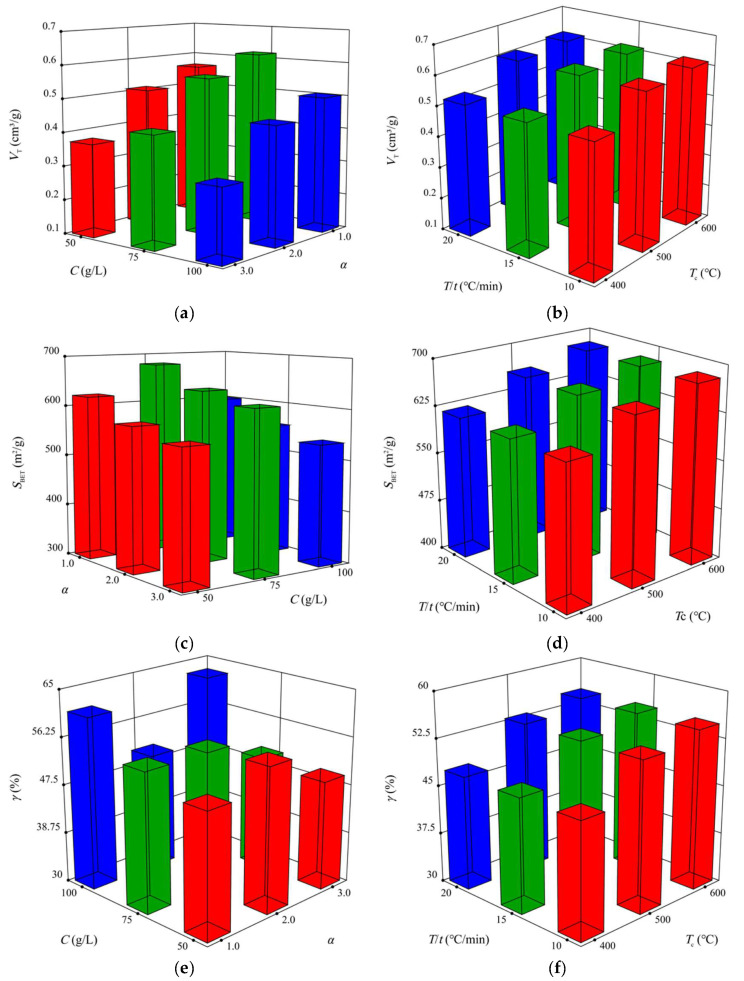
D-optimal design response surfaces for carbonization pore properties. (**a**,**c**,**e**) Coupled impacts of impregnation ratio (*α*) and impregnation concentration (C) on *V*_T_, *S*_BET_, and *γ*, respectively. (**b**,**d**,**f**) Combined influences of carbonization temperature (*T*_c_) and heating rate (*T*/*t*) on *V*_T_, *S*_BET_, and *γ*, respectively. All data were obtained under fixed holding time (120 min) and a carbonization temperature of 600 °C.

To clarify the coupled effects of multiple operational parameters on pore evolution during carbonization, a D-optimal experimental design was adopted (Section 2.2.3). A total of 56 experiments were conducted (Table S1 and Figure 4). Analysis of variance (ANOVA, Table S2) indicated high statistical significance for all three models (*F* = 14.15–32.37, *p* < 0.0001) with insignificant lack of fit (*p* > 0.05), suggesting excellent goodness of fit and robust predictive capability. Pore volume *V* is most sensitive to the impregnation ratio α (*F* = 54.44, *p* < 0.0001), followed by *C* and *T*_c_, indicating that pore expansion is primarily governed by activating agent loading. Specific surface area *S*_BET_ is dominated by impregnation concentration *C* (*F* = 24.83, *p* < 0.0001), as micropore nucleation depends on the dispersion density of ZnCl_2_ and iron salts within the precursor matrix. Burn-off rate *γ* is primarily controlled by *T*_c_ (*F* = 21.80, *p* < 0.0001), with a significant *α*–*C* interaction (*F* = 14.40, *p* < 0.0001), reflecting that mass loss arises from the combined action of thermal pyrolysis and chemical etching. Such sensitivity heterogeneity necessitates multi-objective optimization to reconcile pore development efficiency with carbon yield.

Three-dimensional response surfaces visualize the interactive regulation rules of key parameters. The coupled effect of *α* and *C* follows a distinct inverted U-shaped trend (Figure 4a,c,e): *V_T_* peaks at *α* = 1.0–2.0 and *C* ≈ 75 g/L. Insufficient impregnation leads to incomplete pore development, while an excessively high impregnation ratio (*α* > 2.5) causes severe over-etching and collapse of pore walls. For *S*_BET_, increasing *C* continuously promotes micropore formation within the tested range via intercalation of dispersed Zn and Fe into lignocellulosic fibers; *C* exceeding 100 g/L induces surface salt agglomeration that blocks internal diffusion channels and suppresses deep pore development. Carbonization temperature and heating rate jointly modulate pyrolysis kinetics and pore evolution (Figure 4b,d,f). Raising *T*_c_ from 400 to 600 °C monotonically increases *V_T_*, *S*_BET_, and *γ* by intensifying lignocellulose decomposition, ZnCl_2_-catalyzed aromatization, and in situ Fe_3_O_4_ hard templating. Heating rate exhibits a threshold effect: 10–15 °C/min allows gradual volatile release to construct interconnected pore networks, whereas rates above 15 °C/min form a dense surface char shell that traps internal volatiles and hinders interior pore growth. The extreme run with high *α*, C, and *T*_c_ (Table S1, Run 44) exhibited *γ* = 68.25% and *V* = 0.153 cm^3^/g, directly confirming structural collapse induced by excessive etching.

These trends can be mechanistically interpreted from a multi-stage pore evolution perspective [19]. During impregnation, ZnCl_2_ and FeSO_4_ diffuse into lignocellulosic inter-fiber spaces via capillary action and bind with hydroxyl groups, achieving molecular-level dispersion. At 200–400 °C, sequential decomposition of hemicellulose and cellulose releases volatiles to carve primary pore channels; ZnCl_2_ exerts a dehydrative carbonization effect to suppress tar formation, while partial Fe^2+^ oxidizes into Fe_3_O_4_ nanocrystals acting as in situ hard templates. At 400–600 °C, lignin degradation and carbon skeleton condensation proceed; gasified ZnCl_2_ continuously etches carbon walls to generate micropores while growing Fe_3_O_4_ grains prop open mesoporous channels, enabling synchronous micropore–mesopore development. Essentially, impregnation parameters regulate agent loading and dispersion, while thermal parameters modulate pyrolysis and condensation kinetics, collectively enabling precise tailoring of the primary porous framework.

Numerical optimization based on regression models yields the global optimum targeting balanced maximization of *V* and *S*_BET_ with controlled burn-off: *α* = 1.0, *C* = 75 g/L, *T*_c_ = 600 °C, heating rate = 15 °C/min, and *t* = 120 min. The model predicts *V_T_* = 0.619 cm^3^/g, S_BET_ = 680 m^2^/g, and *γ* = 55.1% under this condition. Triplicate validation experiments yield measured values of 0.612 ± 0.015 cm^3^/g, 675 ± 23 m^2^/g, and 54.8 ± 1.2%, respectively, with relative errors < 5% for all responses, confirming high predictive accuracy and experimental reproducibility. The optimized carbonized product features a well-developed primary porous framework with a balanced pore composition and moderate carbon yield, serving as a high-quality precursor for the subsequent two-stage activation process.

### 3.2. Activation Optimization and Hierarchical Pore Architecture

#### 3.2.1. Stepwise Activation Synergy and Parameter Optimization

Following carbonization, phosphoric acid impregnation coupled with deep activation via steam was systematically optimized via the BBD to precisely tailor the hierarchical porous architecture, aiming to strike an optimal balance between micropore adsorption capacity and mesopore mass transfer efficiency (Section 2.2.4). A three-factor, three-level experimental matrix was constructed (Table S3). The analysis of variance results (Table S4) confirm that both regression models for *V*_T_ and *S*_BET_ are highly statistically significant (*F* = 12.44 and 22.70; *p* = 0.0016 and 0.0002, respectively), with insignificant lack of fit terms (*p* > 0.05) for both responses. This verifies satisfactory goodness of fit and robust predictive capacity for pore structure evolution throughout the activation process.

**Figure 5:**
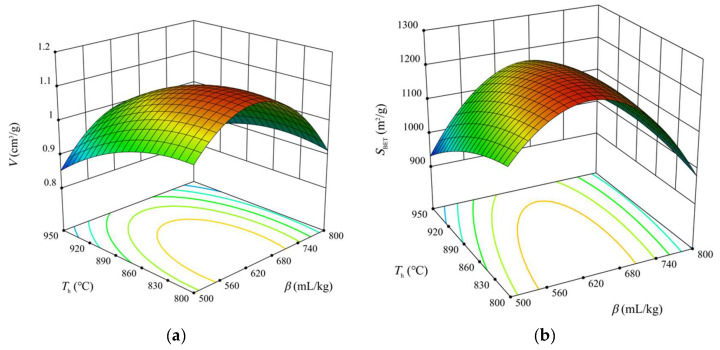
Response surface diagram of the activation stage. Three-dimensional contour response surfaces from 17 Box–Behnken activation experiments depicting the synergistic effects of phosphoric acid impregnation ratio (β) and activation temperature (*T*_h_) under a fixed holding time of 100 min. (**a**) Description of *V_T_*. (**b**) Description of *S*_BET_.

Response surface profiles further unravel the coupled regulation rules governing pore evolution. As shown in Figure 5, activation temperature is identified as the most influential parameter. Increasing Th from 800 to 875 °C enhances pore development by promoting carbon etching and volatile release, while further elevation to 950 °C induces excessive carbon burn-off and pore wall collapse, reducing both pore volume and specific surface area. The phosphoric acid impregnation ratio exhibits a distinct inverted U-shaped effect; the optimal value of 650 mL/kg balances etching intensity and structural integrity. At low ratios, insufficient phosphoric acid results in inadequate chemical etching and underdeveloped pores; at high ratios, excess phosphate species precipitate and block newly formed pores. Activation time peaks at 100 min, with shorter times causing incomplete activation and longer times inducing over-etching and structural degradation.

Multi-objective numerical optimization was performed based on the established regression models, yielding the optimal process parameters for the activation stage: *β* = 650 mL/kg, *T*_h_ = 875 °C, and *t*_h_ = 100 min. As detailed in Table 4, under these conditions, parallel validation experiments yield measured values of 1.123 ± 0.021 cm^3^/g for *V*_T_ and 1244.69 ± 24.35 m^2^/g for *S*_BET_, representing significant improvements over single ZnCl_2_-activated ZYC-Zn and carbons prepared in preliminary work (Table 4).

#### 3.2.2. Hierarchical Pore Architecture and Fine Structural Analysis

Comprehensive textural characterization was subsequently conducted to elucidate the hierarchical pore configuration of ZYC-ZFe (Table 4). Comparison between ZYC-ZFe and ZYC-Zn reveals the multiple regulatory effects of in situ Fe doping on pore structure. ZYC-ZFe achieves a 20.5% higher *S*_BET_ (1244.69 vs. 1057.18 m^2^/g) and 22.9% larger total pore volume (1.123 vs. 0.914 cm^3^/g) than ZYC-Zn, with a nitrogen adsorption capacity of 582.14 cm^3^/g·STP (15.9% higher than ZYC-Zn’s 502.23 cm^3^/g·STP). These results collectively corroborate that in situ Fe doping effectively promotes overall porosity development without inducing severe structural collapse of the carbon matrix.

Further pore size distribution analysis demonstrates a more well-tailored pore hierarchy in ZYC-ZFe. The micropore specific surface area is maintained at 293.7 m^2^/g to reserve sufficient adsorption sites, while the external specific surface area reaches 950.99 m^2^/g, which is 1.8 times that of ZYC-Zn (528.68 m^2^/g). The most prominent difference lies in pore composition: ZYC-ZFe achieves a 43.9% increase in mesopore volume (0.658 vs. 0.457 cm^3^/g) while preserving the microporous adsorption framework in a nearly intact state (0.465 vs. 0.457 cm^3^/g). The mesopore ratio rises to 58.6%, with an average pore diameter of 26.8 Å. Such selective growth of mesopores is a key advantage of the in situ Fe_3_O_4_ templating effect, which alleviates the common problem of micropore loss during conventional activation.

Fine structural analysis via NLDFT and QSDFT further confirms its optimal pore grading (Table 5). In the micropore region, ZYC-ZFe retains a complete micropore size distribution: <0.7 nm micropore volume of 0.182 cm^3^/g, 0.7–1.0 nm narrow micropore volume of 0.203 cm^3^/g (well matched with the kinetic diameter of methanol, 0.38 nm), and 1.0–2.0 nm micropore volume of 0.080 cm^3^/g, which are all comparable to those of ZYC-Zn. In the mesopore region of ZYC-ZFe, the 2–5 nm mesopore volume of 0.285 cm^3^/g and the 5–10 nm mesopore volume of 0.247 cm^3^/g are 58.3% and 49.7% higher than ZYC-Zn, respectively, and the 10–50 nm mesopore volume reaches 0.126 cm^3^/g (12.5% higher than ZYC-Zn), forming continuous rapid diffusion channels.

Additionally, the pore connectivity index of ZYC-ZFe reaches 0.78, which is 20% higher than ZYC-Zn (0.65), significantly reducing dead-end pores and intraparticle diffusion bottlenecks. Its adsorption potential distribution width is 8.2 kJ/mol, which is 26.2% wider than the 6.5 kJ/mol width of ZYC-Zn, indicating a broader energy distribution of surface adsorption sites and stronger affinity for methanol molecules. The iron content of ZYC-ZFe reaches 15.39%, which is 4.9 times that of ZYC-Zn (3.17%), laying a material foundation for the enhancement of thermal conductivity.

Benchmarking against three mainstream adsorbent technical routes demonstrates the comprehensive advantages of ZYC-ZFe. Compared with sludge-based Fe-doped carbons (IM-WNC, PM-WNC), ZYC-ZFe matches the thermal conductivity of in situ doped IM-WNC (4.517 vs. 4.586 W/(m·K)) with 3.1× higher micropore volume (0.465 vs. 0.147 cm^3^/g), resolving the inherent shortage of adsorption sites in high-ash sludge-derived materials. Its Fe content exceeds IM-WNC and PM-WNC by 42.2% and 128.0%, respectively. The poor thermal conductivity of post-impregnated PM-WNC (2.105 W/(m·K)) further confirms the superiority of the in situ doping strategy. Regarding graphene-doped coal-based carbons (GHSCs), despite marginally higher specific surface areas (1343.00 m^2^/g), they exhibit severely imbalanced pore structures: mesopore ratios exceed 75%, and micropore volumes are 37.3% and 38.2% lower than ZYC-ZFe. ZYC-ZFe also delivers 73% higher thermal conductivity (4.517 vs. 2.61 W/(m·K)) and a balanced micropore–mesopore ratio (1:1.4 vs. ~1:3.1), avoiding the pore imbalance and high cost of graphene-doped systems.

Overall, ZYC-ZFe strikes an optimal balance among three core structural metrics: high micropore volume for adsorption, well-developed mesoporous network for mass transfer, and superior thermal conductivity. This hierarchical architecture underpins its strong methanol adsorption–desorption performance, which is verified quantitatively in subsequent sections.

#### 3.2.3. Multifunctional Roles of Fe_3_O_4_ and Surface Chemistry Modulation

The Fe impregnation strategy yields the in situ formation of Fe_3_O_4_ nanoparticles embedded within the carbon matrix. Prior to elaborating its multi-regulatory effects on carbon structure and surface properties, combined FTIR, XPS, and SEM characterizations—using iron-free ZYC-Zn as a blank control—are performed to fully confirm the presence of the spinel Fe_3_O_4_ phase.

**Figure 6:**
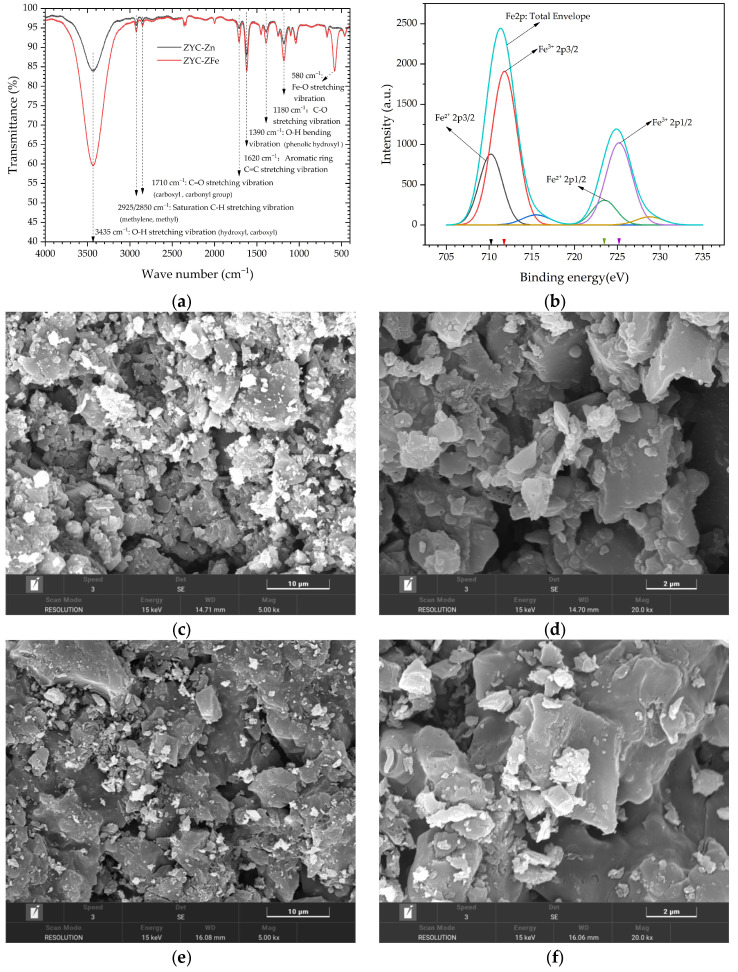
Surface chemical and morphological characterizations of ZYC-ZFe and ZYC-Zn. (**a**) FTIR spectra comparing functional group vibrations of Fe-doped ZYC-ZFe and undoped ZYC-Zn; the characteristic peak at 580 cm^−1^ corresponds to the Fe–O stretching vibration. (**b**) High-resolution XPS Fe 2p spectrum of ZYC-ZFe, deconvoluted to distinguish Fe^2+^ and Fe^3+^ species and confirm the magnetite phase. (**c**,**d**) SEM images of ZYC-ZFe. (**e**,**f**) SEM images of ZYC-Zn.

The FTIR results (Figure 6a, Table S5) show a characteristic absorption band at 570–590 cm^−1^ for ZYC-ZFe, which is assigned to the mixed Fe-O lattice stretching vibration of spinel Fe_3_O_4_; no equivalent band is detected for ZYC-Zn, ruling out intrinsic TCM-residue-derived impurities. The high-resolution Fe 2p spectrum of ZYC-ZFe (Figure 6b) is deconvoluted into six peaks, with fitting parameters listed in Table S5. The total area fraction of Fe^2+^ species (20.3% + 7.1%) reaches 27.4%, while Fe^3+^ species account for 67.5% (44.0% + 23.5%), delivering an Fe^2+^/Fe^3+^ ratio of ~1:2.46, which is close to the theoretical stoichiometric ratio of 1:2 for magnetite Fe_3_O_4_. Two characteristic shake-up satellite peaks appear at 715.5 e*V* and 728.8 e*V*, which exclude zero-valent metallic iron and confirm the coexistence of ferrous and ferric species. The slight deviation from the ideal 1:2 ratio originates from a thin amorphous iron oxyhydroxide layer covering the sample surface, as well as the surface-restricted probing depth of XPS. In addition, the enhanced absorbance of oxygen-containing functional groups (3435, 1710, 1390, and 1180 cm^−1^) on ZYC-ZFe aligns well with XPS O 1s deconvolution and Boehm titration data (Table S7). Boehm titration reveals that ZYC-ZFe bears 2.43 mmol/g total acidic functional groups, which is 33.5% higher than the 1.82 mmol/g measured for ZYC-Zn. Rich carboxyl and phenolic hydroxyl groups can act as anchoring sites for Fe^2+^ precursors and facilitate the in situ crystallization of Fe_3_O_4_ nanoparticles during carbonization.

The Fe-O vibrational fingerprint from FTIR and the quantitative Fe^2+^/Fe^3+^ ratio derived from XPS mutually corroborate the in situ generation of Fe_3_O_4_ nanoparticles within the carbon skeleton. Dispersed Fe_3_O_4_ serves three functions simultaneously: a hard template generating interconnected mesopores, a continuous thermal conduction network, and abundant Lewis acid sites. These components synergistically tune pore architecture, surface polarity, and thermal conductivity, thereby elevating the methanol uptake and refrigeration performance of the composite adsorbent. Low- and high-magnification SEM micrographs (Figure 6c–f) provide supplementary morphological evidence: uniformly dispersed white nanocrystals are visible across the ZYC-ZFe carbon matrix, whereas no such granular precipitates emerge on ZYC-Zn surfaces.

With the crystalline Fe_3_O_4_ phase unambiguously verified, its four synergistic regulatory effects on pore grading, structural stability, surface polarity, and inherent thermal conductivity may be elaborated based on the pore structural parameters (Table 4 and Table 5) and surface characterization data obtained in this section:

(1) Hard template effect toward the construction of hierarchically interconnected mesopores: During carbonization at 400–600 °C, homogeneously dispersed Fe_3_O_4_ nanocrystals occupy the inter-fiber voids within lignocellulosic precursors and suppress thermal shrinkage of the carbon matrix. In the subsequent phosphoric acid etching and two-step deep steam activation process, partial dissolution of the embedded Fe_3_O_4_ replicas generates continuous mesoporous cavities with dimensions consistent with those of the nanoparticles. Relative to ZYC-Zn, ZYC-ZFe exhibits a 43.9% elevation in mesopore volume, alongside an increase in the pore connectivity index from 0.65 to 0.78 (Table 5). Previous investigations [35,36] have documented that this templating mechanism selectively widens mesoscale channels while largely preserving the intact microporous framework, thereby mitigating the pervasive trade-off between micropore collapse and overexpanded pore structures commonly encountered in conventional single-step activation strategies.

(2) Rigid anchoring effect for inhibiting thermal degradation of carbon skeletons: Pristine biomass-derived carbon matrices are prone to shrinkage and pore-wall sintering upon high-temperature activation (800–900 °C). Embedded Fe_3_O_4_ nanocrystals serve as rigid physical anchors to constrain carbon skeleton deformation, permanently safeguarding the narrow micropores (0.7–1.0 nm) generated during carbonization against structural collapse. Such stabilizing effects induced by iron doping on lignocellulosic carbon have been extensively validated. Relevant research [37,38] indicates that iron oxide nanoparticles encapsulated within carbon frameworks function as physical anchors to alleviate matrix shrinkage and pore deterioration over successive thermal cycling treatments.
(3) Generation of Lewis acid sites and enrichment of surface polar functional groups: Fe^3+^ sites on the Fe_3_O_4_ lattice act as abundant Lewis acid sites, which can form polar coordination bonds with the lone electron pairs on oxygen atoms of methanol molecules [39,40]. Meanwhile, embedded Fe_3_O_4_ catalyzes the formation and stabilization of oxygen-containing functional groups across the carbon surface. Boehm titration data (Table S7) reveal that ZYC-ZFe contains 2.43 mmol/g total acidic functional groups, which is 33.5% higher than the 1.82 mmol/g measured for ZYC-Zn, with markedly elevated contents of carboxyl, lactonic, and phenolic hydroxyl groups. The more intense characteristic stretching bands assigned to O–H, C=O, and C–O vibrations in the FTIR spectra of ZYC-ZFe (Figure 6a) further verify the enrichment of polar surface moieties. These polar functional groups establish hydrogen-bonding interactions with methanol molecules to enhance adsorption affinity. Moreover, Fe^3+^ species coordinate with surface oxygenated groups and suppress the thermal decomposition of functional groups at elevated temperatures. The point of zero charge (*p*H_pzc_) of ZYC-ZFe is 5.8 ± 0.2, which is lower than the value of 6.5 ± 0.2 obtained for ZYC-Zn, demonstrating a more negatively charged surface under near-neutral environments. Such negative surface charge reinforces electrostatic attraction toward polar methanol molecules and boosts surface hydrophilicity, thereby promoting methanol vapor condensation and capillary filling inside micropores.
(4) Thermal conductivity enhancement: Tian et al. [41] quantitatively confirmed that iron oxide modification establishes continuous thermal conduction channels within carbon matrices, substantially elevating the thermal conductivity of biomass-derived carbon. In the study, interconnected Fe_3_O_4_ nanoparticles formed uninterrupted thermal transport pathways embedded throughout the carbon skeleton. The measured thermal conductivity of ZYC-ZFe is 4.517 W/(m·K), representing a 44.7% improvement over the 3.122 W/(m·K) recorded for ZYC-Zn. Elevated thermal conductivity lowers thermal resistance and boosts the energy efficiency of adsorption refrigeration systems. More crucially, the simultaneous promotion of thermal conductivity and mass transfer efficiency alleviates the intrinsic performance trade-off widely observed in conventional porous carbon adsorbents [42,43].

In summary, multidimensional characterizations corroborate the in situ formation of homogeneously dispersed spinel Fe_3_O_4_ nanoparticles embedded in the TCM residue carbon matrix. Fe_3_O_4_ exerts synergistic multifunctional modulation: hard templating constructs a highly interconnected micropore–mesopore network; rigid physical anchoring stabilizes the carbon skeleton against thermal collapse; Fe^3+^ Lewis sites together with abundant polar functional groups tune surface adsorption affinity; and interconnected Fe_3_O_4_ crystallites establish uninterrupted thermal conduction pathways. Collectively, these coordinated effects overcome four intrinsic limitations of pristine herbal biochar: ill-proportioned micropore–mesopore distribution, weak thermal structural stability, scarce surface active sites, and inferior inherent thermal conductivity. This achieves integrated optimization of the porous architecture, surface chemistry, and thermal performance of the resulting hierarchical carbon adsorbent.

### 3.3. Methanol Adsorption–Desorption Performance

#### 3.3.1. Adsorption Isotherm and Equilibrium Adsorption Capacity

**Figure 7:**
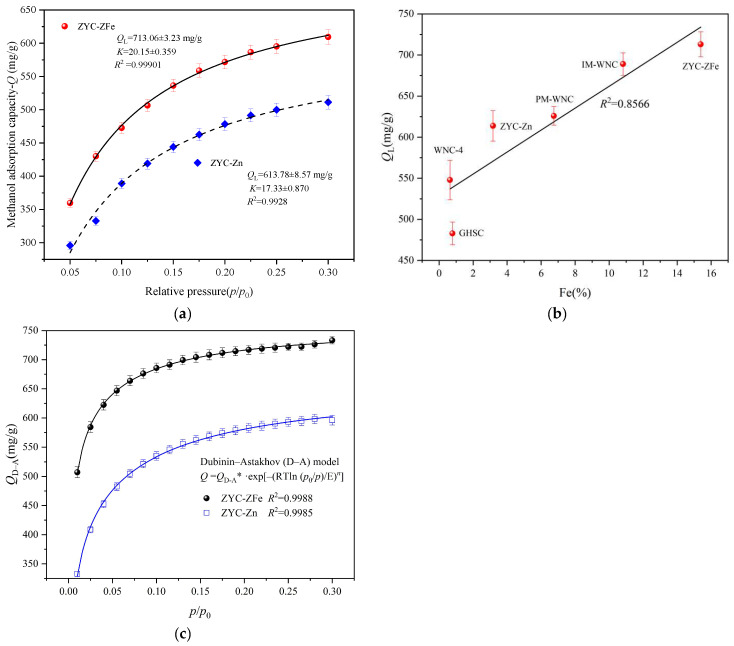
Methanol adsorption isotherms and the correlation of iron content with adsorption capacity. (**a**) Langmuir model fitting curves of methanol adsorption equilibrium data for ZYC-ZFe and ZYC-Zn; (**b**) linear fitting plot revealing the positive correlation (*R*^2^ = 0.8566) between iron mass fraction and Langmuir saturated methanol adsorption capacity; and (**c**) Dubinin–Astakhov (D–A) adsorption isotherms of two carbon samples, with fitting coefficients (*R*^2^ > 0.99) for both materials.

Building on the synergistic advantages of the hierarchical pore structure and surface chemical properties discussed above, methanol adsorption isotherms of ZYC-ZFe and the undoped control sample ZYC-Zn were measured within the typical operating range of adsorption refrigeration systems (25 °C, *p*/*p*_0_ = 0.05–0.3). The experimental data were fitted using two classical adsorption models: the Langmuir model and the Dubinin–Astakhov (D–A) model, with the results summarized in Table 6 and Figure 7.

At low relative pressure (*p*/*p*_0_ = 0.05), ZYC-ZFe achieved a methanol uptake of 359.81 mg/g, representing a 21.63% increase compared to ZYC-Zn (295.87 mg/g). This pronounced enhancement under dilute vapor conditions is attributed to the enriched polar oxygen-containing functional groups (total acidic group content of 2.43 mmol/g, which is 33.5% higher than ZYC-Zn) and uniformly dispersed Fe^3+^ Lewis acid sites on the surface of ZYC-ZFe. As the relative pressure increased, the gap in adsorption capacity between the two samples widened further, reaching a maximum difference of 76.74 mg/g at *p*/*p*_0_ = 0.07. This is attributed to the well-developed hierarchical pore structure of ZYC-ZFe. The maximum Langmuir adsorption capacity (*Q*_L_*) of ZYC-ZFe reached 713.06 ± 15.23 mg/g, which is 16.18% higher than that of ZYC-Zn (613.78 ± 18.57 mg/g). Combined with the structural analysis in the previous section, ZYC-ZFe fully retains all microporous adsorption sites while effectively reducing mass transfer resistance through the interconnected mesopore network, achieving efficient utilization of adsorption capacity. As illustrated in Figure 7b, the Langmuir adsorption capacity increases linearly with the iron content of biochar samples (*R*^2^ = 0.8566). Higher Fe loading brings abundant Fe^3+^ active sites and well-developed mesoporous networks, jointly enhancing methanol capture ability, which directly confirms the critical promotion effect of in situ Fe doping on adsorption performance.

Benchmarking against nine representative state-of-the-art and commercial adsorbents (Table 6) confirms the industry-leading equilibrium adsorption capacity of ZYC-ZFe, with comprehensive advantages across all technical routes. Regarding the sludge-based Fe-doped carbons, ZYC-ZFe delivers 3.5%, 13.9%, and 29.0% higher maximum adsorption capacity than in situ doped IM-WNC (688.99 mg/g), post-impregnated PM-WNC (625.98 mg/g), and pristine sludge carbon WNC-4 (552.67 mg/g), respectively. Despite matching thermal conductivity, IM-WNC retains only 32.3% of ZYC-ZFe’s micropore volume, severely constraining adsorption sites and capacity limits. PM-WNC gains minimal performance improvement, as Fe nanoparticle agglomeration precludes both effective heat conduction pathways and pore structure optimization. Regarding the graphene-doped coal-based carbons, ZYC-ZFe outperforms the GHSC (534.94 mg/g) by 33.3%. Despite the larger specific surface area of the GHSC (1343.00 m^2^/g), highly skewed pore structures (mesopore ratio > 75%, deficient micropores) prevent most of the surface area from converting into valid methanol adsorption sites, which is compounded by high graphene doping costs.

It is critical to clarify the limitations of Langmuir fitting for biomass-derived porous carbons. As shown in Table 4 and Figure 6, ZYC-ZFe contains abundant heterogeneous adsorption sites, including Fe^3+^ Lewis acid sites, variable oxygen-containing polar functional groups, and multi-scale micro/mesopores with different confinement effects, which breaks the Langmuir hypothesis of identical, non-interactive adsorption sites. Surface energy heterogeneity data (Table 5, adsorption potential distribution width = 8.2 kJ/mol) further verifies the non-uniformity of the carbon surface. Therefore, we avoid drawing absolute mechanistic conclusions of monolayer adsorption merely from Langmuir fitting results. D–A model fitting further clarifies the adsorption mechanism advantages of ZYC-ZFe. Its maximum adsorption capacity (*Q*_D-A_^*^) reached 744.11 ± 1.55 mg/g, with only 4.3% deviation from the Langmuir result, verifying measurement reliability. The characteristic adsorption energy *E* of ZYC-ZFe was 17.55 ± 0.17 kJ/mol, which was 23.7% higher than that of ZYC-Zn (14.19 ± 0.11 kJ/mol). A larger characteristic adsorption energy reflects deeper adsorption potential wells for methanol molecules, which demonstrates stronger intermolecular binding affinity between methanol and the carbon surface. This trend agrees with the higher low-pressure adsorption uptake of ZYC-ZFe and further verifies the enhanced adsorption interaction originating from abundant Fe^3+^ Lewis acid sites and enriched surface oxygenated groups. With a higher *n* value (2.20 ± 0.04 vs. 1.95 ± 0.05), ZYC-ZFe shows more uniform surface energy distribution homogenized by in situ Fe_3_O_4_, narrowing inter-site energy gaps for more stable and efficient adsorption.

Notably, multiple reference adsorbents exhibit a decoupled trend between apparent *S*_BET_ and equilibrium methanol uptake. The GHSC achieves a higher apparent *S*_BET_ (1343 m^2^/g) than ZYC-ZFe yet delivers far inferior methanol adsorption capacity (534.94 mg g^−1^ vs. 713.06 mg g^−1^). This universal contradiction across different carbon systems proves that BET surface area alone cannot quantify the number of effective adsorption sites for polar methanol molecules. The superior methanol capture performance of ZYC-ZFe is primarily governed by well-matched micropore–mesopore hierarchical structure, abundant Fe^3+^ Lewis acid sites, and enriched surface oxygen-containing polar functional groups; a high *S*_BET_ only serves as a secondary auxiliary structural characteristic, rather than a decisive performance-determining factor. As proposed recently by Hamieh [44], a two-dimensional thermodynamic adsorption framework was established to resolve systematic deviations originating from the three idealized hypotheses of classical BET theory. Thus, BET surface area should be interpreted as an apparent parameter rather than an absolute geometric quantity, and this theory perfectly rationalizes the decoupled trend between apparent *S*_BET_ and actual methanol uptake observed in the comparative adsorbents.

#### 3.3.2. Adsorption Kinetics and Mass Transfer Characteristics

**Figure 8:**
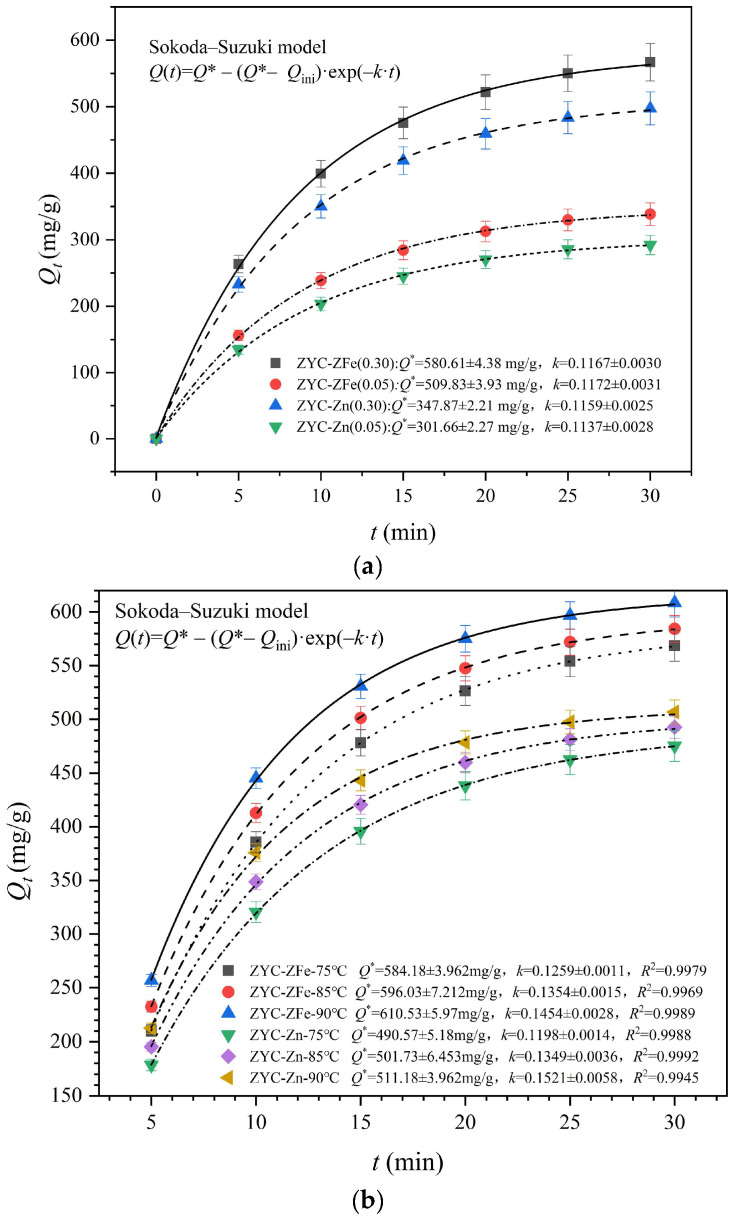
Kinetic curves fitted by the Sokoda–Suzuki model. Time-dependent methanol uptake curves for adsorption and desorption processes of ZYC-ZFe and ZYC-Zn. (**a**) Adsorption kinetic profiles measured at 15–25 °C; (**b**) desorption kinetic profiles tested at 75–90 °C. The statistical analysis indicates significant differences (*p* < 0.05) in mass transfer performance between the two carbon materials for all paired comparisons.

Adsorption and desorption kinetic curves were fitted by the widely recognized Sokoda–Suzuki model to quantitatively characterize the internal mass transfer resistance and dynamic adsorption/desorption rate of carbon materials (Figure 8). The key kinetic parameters, including equilibrium adsorption capacity (Q*) and surface diffusion coefficient (D_S0_), at different temperatures are summarized in Table 7.

At adsorption temperatures of 15, 20, and 25 °C, the equilibrium adsorption capacity Q* of ZYC-ZFe reached values of 580.61, 552.34, and 526.47 mg/g, respectively, which were significantly higher than ZYC-Zn (374.87, 356.21, 338.95 mg/g). The surface diffusion coefficient *D*_S0_ of both samples increased with the rise of adsorption temperature, which was attributed to the enhanced thermal motion of methanol molecules and reduced diffusion activation barrier at higher temperature. At 25 °C, the *D*_S0_ of ZYC-ZFe was 1.8 × 10^−10^ m^2^/s, while that of ZYC-Zn was only 1.1 × 10^−10^ m^2^/s; the surface diffusion capacity of ZYC-ZFe was improved by 63.6%. At an adsorption temperature of 15 °C, ZYC-ZFe delivers a surface diffusion coefficient (*D*_S0_ = 1.4 × 10^−10^ m^2^/s) far exceeding ZYC-Zn’s value of 0.87 × 10^−10^ m^2^/s. This result verifies ZYC-ZFe’s stable fast mass transfer performance under low-temperature adsorption operating conditions. Under the test condition of *p*/*p*_0_ = 0.3, ZYC-ZFe reached 97.6% of equilibrium adsorption capacity within 30 min, and 83.9% of saturated adsorption capacity was completed in the first 15 min. ZYC-Zn only achieved 87.8% equilibrium capacity after 30 min of adsorption. The prominent kinetic advantage of ZYC-ZFe originates from its optimized hierarchical pore system: abundant interconnected 2–10 nm mesopores act as high-speed diffusion channels for methanol vapor, greatly shortening the transmission path from the external particle surface to internal microporous adsorption sites and reducing intraparticle mass transfer resistance. Fast adsorption kinetics constitute a core advantage of industrial adsorption refrigeration, which supports the design of short-period systems and effectively lifts the overall specific cooling power to break the low-power bottleneck of traditional activated carbon adsorbents.

Desorption dynamic tests were carried out at 75, 85, and 90 °C to match the typical low-grade waste heat temperature range of adsorption refrigeration. As shown in Figure 8b and Table 7, the equilibrium desorption capacity and surface diffusion coefficient of both samples increased synchronously with desorption temperature. At the target regeneration temperature of 90 °C, ZYC-ZFe possessed a desorption equilibrium capacity of 610.53 mg/g and a surface diffusion coefficient of 1.98 × 10^−10^ m^2^/s, far exceeding ZYC-Zn (511.18 mg/g, 1.32 × 10^−10^ m^2^/s). The elevated desorption diffusion coefficient indicated that methanol molecules could rapidly escape from the carbon pore channels under low-temperature heating, and the material realized efficient regeneration below 100 °C. The enriched Lewis acid sites and polar oxygen-containing functional groups introduced by in situ Fe doping moderately reduced the adsorption binding energy between methanol and the carbon matrix, lowering the desorption activation energy without sacrificing equilibrium adsorption capacity.

Meanwhile, the high-connectivity hierarchical pore network eliminates a large number of dead-end pores, ensuring that desorbed methanol vapor can be quickly exported out of the adsorbent bed, further accelerating the desorption rate and improving the effective utilization ratio of adsorbents in cyclic operation.

#### 3.3.3. Thermodynamic Analysis

To gain insight into the thermodynamic driving forces governing methanol adsorption–desorption on the carbon adsorbents, the Gibbs free energy change (Δ*G*), enthalpy change (Δ*H*), and entropy change (Δ*S*) were calculated. The thermodynamic parameters were derived from the temperature-dependent adsorption equilibrium data using the van’t Hoff equation and the Gibbs–Helmholtz relationship, and the results are summarized in Table 8.

The adsorption Gibbs free energy (Δ*G*ads) of ZYC-ZFe ranged from −9.90 to −7.46 kJ/mol over 288.15–298.15 K, which is consistently more negative than ZYC-Zn (−7.23 to −5.20 kJ/mol), confirming a stronger thermodynamic driving force for methanol adsorption. The adsorption enthalpy (Δ*H*_ads_) of ZYC-ZFe was −38.72 kJ/mol, which is 20.4% higher than ZYC-Zn (−32.15 kJ/mol), indicating a mixed physisorption–chemisorption mechanism enhanced by Fe^3+^ Lewis acid sites and polar oxygen groups. This moderate ΔHads value ensures adequate methanol uptake while enabling efficient regeneration at ≤100 °C. The negative adsorption entropy (Δ*S*_ads_) ranged from −99.9 to −104.8 J/(mol·K)^−1^ for ZYC-ZFe, which is more negative than ZYC-Zn (−86.4 to −90.4 J/(mol·K)^−1^), reflecting greater orientational constraints of methanol molecules within ZYC-ZFe’s more confined and energetically heterogeneous pore network. For desorption, positive ΔGdes values (0.67–1.39 kJ/mol at 348.15–363.15 K) confirmed the non-spontaneous, endothermic nature that requires external heat input. ZYC-ZFe exhibited higher ΔS_des_ (104.8 vs. 90.4 J/(mol·K)^−1^), reflecting greater entropy gain as methanol escaped from its confined pore structure. The adsorption and desorption activation energies (*E*a = 10.2 kJ/mol, *E*_d_ = 12.8 kJ/mol) were both modest, confirming that surface diffusion is not rate-limiting and that low-temperature regeneration (90 °C) is thermodynamically feasible. The thermodynamic synergy—moderately enhanced Δ*H*_ads_ for robust uptake, low *E*_d_ for efficient regeneration, and a strong entropic driving force for desorption—establishes the energetic foundation for ZYC-ZFe’s superior cyclic refrigeration performance.

#### 3.3.4. Long-Term Cyclic Stability and Structural Durability

**Figure 9:**
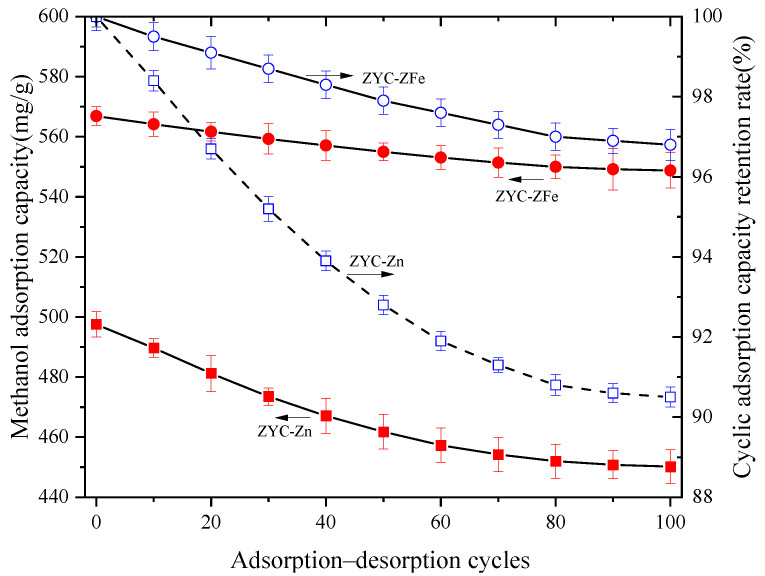
Cyclic adsorption stability over 100 adsorption–desorption cycles. Variation of saturated methanol adsorption capacity of ZYC-ZFe and ZYC-Zn during 100 consecutive cycles (adsorption at 23 °C, desorption at 90 °C). Statistical analysis confirms significant differences (*p* < 0.05) in cyclic capacity retention between the two adsorbents for all paired comparisons.

Long-term cyclic stability is a critical criterion for practical adsorption refrigeration applications, as repeated thermal cycling and adsorbate loading–unloading often induce structural degradation and active site loss. A 100-cycle adsorption–desorption test was conducted under realistic operating conditions (30 min cycle, 23 °C adsorption, and 90 °C desorption) (Figure 9).

After 100 continuous adsorption–desorption cycles, ZYC-ZFe only loses 3.2% of its initial saturated methanol adsorption capacity. The initial minor drop (first 30 cycles) is attributed to the removal of weakly bound surface species and minor structural rearrangement; after which, the performance stabilized with an average decay rate of less than 0.03% per cycle. Post-cycle characterization confirmed the excellent structural and chemical integrity of ZYC-ZFe. It retained 96.5% of its original specific surface area, 98.5% of micropore volume, and 97.1% of mesopore volume. The iron content remained at 97.6%, and total acidic functional groups were preserved at 95%, verifying the robust anchoring effect of Fe^3+^. Fe^3+^ forms strong coordination bonds with carboxyl and hydroxyl groups, preventing active site leaching and pore collapse during thermal cycling. This mechanism effectively resolves the dual challenges of pore degradation and site loss that plague conventional biomass carbons, ensuring long-term operational reliability and cost-effectiveness.

### 3.4. Comprehensive Refrigeration Performance

#### 3.4.1. Dynamic Heat Transfer Characteristics of the Adsorbent Bed

**Figure 10:**
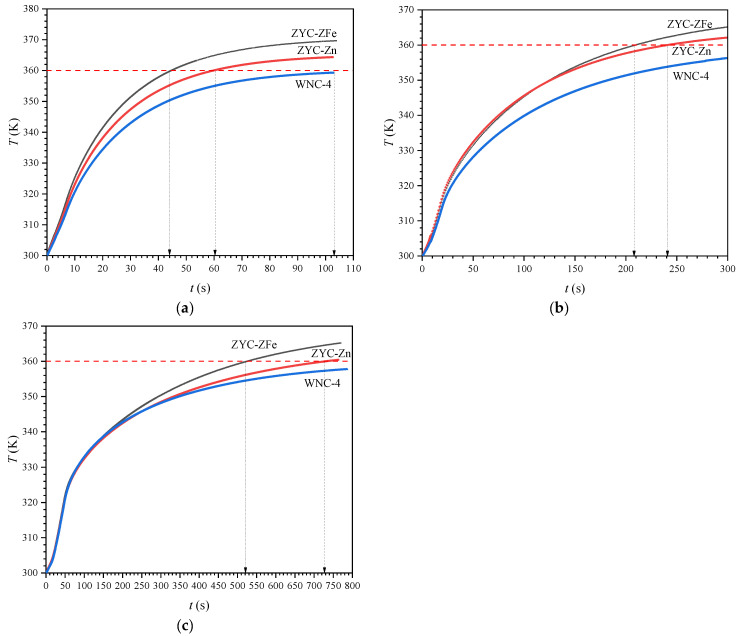
CFD-simulated transient temperature profiles of adsorbent beds with variable sizes. Central bed temperature rise curves obtained via transient CFD simulations under a constant heating water temperature of 370 K for cylindrical adsorbent beds with inner diameters of (**a**) 100 mm, (**b**) 200 mm, and (**c**) 400 mm.

Three adsorbent beds (100, 200, and 400 mm inner diameter) were evaluated via CFD simulation to quantify the scale-up effect on dynamic heat transfer (Figure 10a and Figure S1). The simulation setup, governing equations, and boundary conditions are described in Section 2.6. As the bed size increases, extended radial heat paths raise overall thermal resistance, universally prolonging heating time and worsening temperature uniformity—the core bottleneck restricting industrial deployment of conventional porous carbon adsorbents.

ZYC-ZFe consistently outperformed both ZYC-Zn and commercial WNC-4 across all scales, and its performance advantage widened with bed enlargement. At the 100 mm small-bed scale (solid-phase conduction-dominated), ZYC-ZFe reached the 360 K regeneration target in ~45 s (vs. ~60 s for ZYC-Zn and >100 s for WNC-4), with minimal radial temperature gradient and no low-temperature stagnant zones, which is attributed to its continuous solid heat conduction network. At the 200 mm medium scale (coupled solid conduction and gas convection), ZYC-ZFe achieved the target in 210 s (40 s and ~90 s faster than controls) with negligible uniformity degradation; its highly connected hierarchical pores reduce fluid resistance and strengthen convective heat transfer to flatten temperature gradients. At the 400 mm industrial scale, both controls required >720 s with severe central low-temperature dead zones, while ZYC-ZFe completed full-bed heating in only 520 s (27% time reduction) and maintained uniform temperature distribution, effectively breaking the radial heat transfer bottleneck of large beds.

The heat transfer differences among samples originate from their distinct microstructures. In situ generated Fe_3_O_4_ nanoparticles in ZYC-ZFe are uniformly dispersed in the carbon matrix and interconnected to form a three-dimensional continuous, high-efficiency solid-phase heat conduction network, greatly enhancing intrinsic thermal conductivity [45]. Meanwhile, acting as a hard template, Fe_3_O_4_ constructs a hierarchically porous structure with high micropore–mesopore connectivity, optimizing gas-phase convective heat transfer on the basis of enhanced solid-phase conduction [29,46]. The coupling of these two effects endows ZYC-ZFe with faster thermal response and superior temperature uniformity across all three bed sizes.

#### 3.4.2. Unit Cooling Capacity and Specific Cooling Power

Building on the excellent dynamic heat transfer characteristics of the adsorbent bed, the actual refrigeration performance of ZYC-ZFe was evaluated under a standard 30 min operating cycle and systematically compared with nine representative state-of-the-art and commercial adsorbents (Table 9). The effective contribution ratio (η), defined as the ratio of actual desorption capacity to saturated adsorption capacity, directly reflects adsorbent utilization efficiency in practical cyclic operation. ZYC-ZFe achieved an effective contribution ratio of 89.20%, which is significantly higher than all reference samples. This outstanding performance stems from its 97.6% desorption efficiency at 90 °C and excellent bed temperature uniformity, which eliminates low-temperature dead zones and ensures that nearly all adsorbent particles participate in the complete adsorption–desorption cycle. In contrast, undoped ZYC-Zn only reached 76.50% due to lower thermal conductivity and slower desorption kinetics, while commercial sludge carbon WNC-4 showed the lowest value (55.81%), as severe thermal lags in the bed center caused incomplete desorption of a large fraction of adsorbents.

Unit cooling capacity per unit mass of adsorbent (*Q*_ref_) is a direct indicator of system refrigeration output. ZYC-ZFe delivered a unit cooling capacity of 812.4 ± 12.5 kJ/kg, which is 1.38 times that of ZYC-Zn (590.7 ± 9.8 kJ/kg), 1.62 times that of in situ iron-doped sludge carbon IM-WNC (501.43 ± 10.03 kJ/kg), and 2.88 times that of commercial WNC-4 (281.75 ± 8.07 kJ/kg). This improvement is attributed to the combined effects of its high saturated adsorption capacity (713.06 mg/g) and high effective utilization ratio. Notably, although the graphene-doped coal-based carbon (GHSC) has a higher specific surface area than ZYC-ZFe, its severely imbalanced pore structure results in lower effective adsorption capacities and effective contribution ratios, leading to unit cooling capacities of only 441.05 kJ/kg—45.7% lower than ZYC-ZFe.

Specific cooling power (*N*_ref_), which characterizes refrigeration output per unit time, is the most critical parameter for evaluating the industrial application potential of adsorption refrigeration systems, especially under short-cycle conditions [24]. Under the 30 min standard cycle, ZYC-ZFe achieved a higher specific cooling power of 1534.6 ± 24.3 kJ/(kg·h). This value is 49.6% higher than that of ZYC-Zn (1025.8 ± 17.2 kJ/(kg·h)), 57.6% higher than SC-GC (973.86 ± 15.28 kJ/(kg·h)), and 92.1% higher than IM-WNC (799.06 ± 15.98 kJ/(kg·h)). This exceptional performance is mainly attributed to its fast adsorption–desorption kinetics (surface diffusion coefficient 60.9% higher than ZYC-Zn) and rapid thermal response, which enable the system to complete a full adsorption–desorption cycle within 30 min without sacrificing capacity. In industrial applications, high specific cooling power means significantly reduced adsorbent loading and bed volume, thereby lowering system initial investment and operating costs.

#### 3.4.3. Coefficient of Performance (COP)

The coefficient of performance (*COP*), defined as the ratio of cooling capacity to desorption heat input, is the core index for evaluating the energy efficiency of adsorption refrigeration systems, which directly determines the economic feasibility of low-grade waste heat utilization [11,25]. To comprehensively assess the applicability of ZYC-ZFe under different low-grade heat source conditions, *COP* values of ZYC-ZFe and nine typical adsorbents were measured at desorption temperatures ranging from 60 °C to 150 °C (Table 10).

As expected, *COP* increased with desorption temperature for all samples, as higher temperatures enhance desorption efficiency and increase effective cyclic adsorption capacity. However, ZYC-ZFe exhibited significant energy efficiency advantages across the entire temperature range, particularly in the 60–90 °C low-temperature interval—corresponding to the temperature range of most industrial waste heat and solar energy resources. At the typical industrial waste heat temperature of 90 °C, ZYC-ZFe achieved a *COP* of 0.52 ± 0.03, which is 1.37 times that of ZYC-Zn (0.38 ± 0.02) and 1.93 times that of WNC-4 (0.27 ± 0.02). Even at a lower desorption temperature of 70 °C, ZYC-ZFe still maintained a *COP* of 0.42, while WNC-4 only reached 0.09, indicating that ZYC-ZFe can effectively utilize low-temperature waste heat resources inaccessible to conventional adsorbents.

The superior low-temperature energy efficiency of ZYC-ZFe originates from three key factors: (1) its moderate adsorption enthalpy (−38.72 kJ/mol) balances adsorption affinity and desorption energy consumption, avoiding the high desorption energy requirement of strongly adsorptive materials; (2) its high thermal conductivity significantly reduces adsorbent bed heat transfer losses and improves input heat utilization efficiency; and (3) the hierarchically porous structure ensures rapid mass transfer, enabling complete desorption at lower temperatures. Compared with traditional zeolite adsorbents, which typically require desorption temperatures above 150 °C to achieve a *COP* > 0.5, ZYC-ZFe reaches a *COP* of 0.52 at only 90 °C, greatly expanding the application scenarios of adsorption refrigeration systems and improving their economic competitiveness.

A comparison of adsorbents based on different technical routes reveals that: The sludge-based iron-doped carbon series (IM-WNC, PM-WNC, and WNC-4) generally has low *COP* in the low-temperature section; this is mainly due to insufficient micropore volume, which leads to small effective cyclic adsorption capacity. Moreover, the series exhibits low thermal conductivity of WNC-4 and PM-WNC, which results in large heat transfer losses. The graphene-doped coal-based carbon series (GHSC, SC-GC) shows rapid *COP* increase in the high-temperature section (120–150 °C) but poor low-temperature performance, which is due to severely imbalanced pore structures and incomplete desorption at low temperatures.

Overall, ZYC-ZFe achieves an optimal balance between specific cooling power and *COP* over a wide temperature range, demonstrating comprehensive advantages over mainstream adsorbents and providing a promising material basis for the industrial application of low-grade waste heat-driven adsorption refrigeration technology.

## 4. Conclusions

This work develops a scalable, eco-friendly strategy to fabricate in situ Fe-doped hierarchically porous carbon (ZYC-ZFe) from high-yield TCM residues for methanol adsorption refrigeration, enabling both high-value utilization of herbal waste and performance enhancement of refrigeration adsorbents. The main conclusions are as follows:

1. The binary precursor (55 wt% *Astragalus membranaceus* residue + 45 wt% *Glycyrrhiza uralensis* residue) exhibits favorable lignocellulosic synergy, achieving an optimal balance among cellulose-supported micropore skeletons, hemicellulose-derived mesopores, and lignin-reinforced structural stability. Process optimization via combined D-optimal and Box–Behnken designs yields ZYC-ZFe with a total pore volume of 1.123 cm^3^/g, and a well-matched micropore–mesopore ratio of 1:1.4.
2. In situ generated Fe_3_O_4_ exerts multifunctional synergistic regulatory effects: it acts as a hard template to form interconnected mesopores (20% higher pore connectivity), enhances long-cycle structural stability, provides dispersed Fe^3+^ Lewis acid sites for improved methanol affinity, enriches surface polar oxygen groups by 33.5%, and elevates thermal conductivity to 4.517 W/(m·K). ZYC-ZFe thus delivers 713.06 mg/g methanol adsorption capacity, 97.6% desorption efficiency at 90 °C within 30 min, and only 3.2% capacity loss after 100 consecutive cycles.
3. The excellent heat and mass transfer properties translate into outstanding refrigeration performance. In 400 mm industrial-scale adsorption beds (CFD simulation), it reduces the core temperature rise time by 27.8% relative to commercial adsorbents, and achieves a specific cooling power of 1534.6 kJ/(kg·h) under 30 min short cycles. At 90 °C, its *COP* reaches 0.52–1.37 times the undoped control value and 1.93 times the commercial sludge carbon values. With stable energy efficiency across 60–150 °C desorption temperatures, the material shows promising potential for low- and medium-grade waste heat utilization.

## Figures and Tables

**Table 1:** Chemical composition of TCM residues (dry basis, wt%).

Component	*Astragalus membranaceus*	*Glycyrrhiza uralensis*	*Pueraria lobata*	*Angelica sinensis*
Cellulose	43.82% ± 2.12%	35.56% ± 1.12%	28.95% ± 1.81%	25.26% ± 2.11%
Hemicellulose	16.35% ± 1.26%	25.21% ± 2.24%	15.26% ± 1.22%	12.25% ± 1.01%
Lignin	15.15% ± 2.11%	20.12% ± 1.05%	15.18% ± 2.78%	14.38% ± 2.02%
Polysaccharide	11.36% ± 0.81%	7.87% ± 0.55%	10.26% ± 0.58%	13.15% ± 0.91%
Crude protein	8.92% ± 0.55%	7.25% ± 0.91%	9.02% ± 0.42%	15.10% ± 1.02%

**Table 2:** Independent variables and level settings in the carbonization–activation stage.

Stage	Factors	Symbol	Responses	Symbol
D-optimal experiment for the carbonization stage	Impregnation ratio	*α*	Total pore volume (cm^3^/g)	*V* *_T_*
	Impregnation concentration (g/L)	*C*	Specific surface area (m^2^/g)	*S*_BET_
	Carbonization temperature (°C)	*T*	Burn-off rate (%)	*γ*
	Temperature rise rate (°C/min)	*T/t*		
	Carbonization time (min)	*t*		
Box–Behnken experiment for the activation stage	Phosphoric acid solution impregnation ratio (mL/kg)	*β*	Total pore volume (cm^3^/g)	*V* *_T_*
	Activation temperature (°C)	*T*	Specific surface area (m^2^/g)	*S*_BET_
	Activation holding time (min)	*t*	Burn-off rate (%)	*γ*

**Table 3:** Pore structure properties of carbonized products from single and blended TCM residues.

TCM Residues	TLC (wt%) ^1^	*V_T_* (cm^3^/g)	*S*_BET_ (m^2^/g)
AM	75.32% ± 2.05%	0.404 ± 0.011	524.26 ± 9.85
GU	80.89% ± 1.98%	0.472 ± 0.009	572.98 ± 8.62
PL	59.39% ± 2.21%	0.319 ± 0.013	427.88 ± 10.24
AS	51.89% ± 2.16%	0.267 ± 0.010	386.27 ± 9.17
75% AM + 25% GU	76.71% ± 2.01%	0.411 ± 0.012	548.36 ± 8.93
55% AM + 45% GU	77.83% ± 1.95%	0.461 ± 0.008	562.17 ± 7.56
45% AM + 55% GU	78.38% ± 1.92%	0.450 ± 0.010	538.25 ± 8.29
25% AM + 75% GU	79.50% ± 1.96%	0.425 ± 0.011	540.25 ± 8.71

^1^ The total lignocellulose content (TLC) = cellulose + hemicellulose + lignin.

**Table 4:** Pore structure distribution and physicochemical property parameters of activated carbon samples.

Parameters		ZYC-ZFe *	ZYC-Zn	IM-WNC [19]	PM-WNC [19]	WNC-4 [28]	GHSC [27]
*Q*_N_	cm^3^/g·STP	582.14 ± 11.23	502.23 ± 9.85	482.44 ± 8.62	396.93 ± 7.54	500.27 ± 9.12	838.48
*S*_BET_	m^2^/g	1244.69 ± 24.35	1057.18 ± 21.26	1122.29 ± 19.87	927.35 ± 17.65	1011.09 ± 22.48	1343.00 ± 26.86
*S*_mi_	m^2^/g	293.7 ± 5.82	528.5 ± 10.24	376.94 ± 7.25	350.41 ± 6.87	/	/
*S*_ex_	m^2^/g	950.99 ± 18.56	528.68 ± 10.32	785.34 ± 15.62	586.93 ± 12.45	/	/
*V*_T_	cm^3^/g	1.123 ± 0.021	0.914 ± 0.018	0.6608 ± 0.012	0.5664 ± 0.010	0.6960 ± 0.013	1.2074 ± 0.024
*V*_mi_	cm^3^/g	0.465 ± 0.009	0.457 ± 0.008	0.1471 ± 0.003	0.1243 ± 0.002	0.1641 ± 0.003	0.2916 ± 0.006
*V*_mes_	cm^3^/g	0.658 ± 0.012	0.457 ± 0.009	0.5037 ± 0.010	0.4421 ± 0.008	0.5319 ± 0.011	0.9158 ± 0.018
*R*_mes_ ^1^	%	58.6 ± 1.2	50.0 ± 1.0	76.2 ± 1.5	78.1 ± 1.6	76.4 ± 1.5	75.8
*D* ^2^	Å	26.8 ± 0.5	25.5 ± 0.4	23.65 ± 0.35	25.95 ± 0.42	22.99 ± 0.32	36.92
*h_f_* ^3^	%	19.25 ± 2.60	15.11 ± 2.31	15.34 ± 3.65	12.11 ± 2.34	5.93 ± 0.45	/
λ	W/(m·K)	4.517	3.122	4.586	2.105	0.892	2.20
Fe	%	15.39	3.17	10.82	6.75	0.64	0.79

^1^ Mesopore ratio. ^2^ Average pore diameter. ^3^ Ash content. Statistical significance was assessed by two-tailed independent sample *t*-test with equal variance assumption, *n* = 5 for each group; * *p* < 0.05 indicates a significant difference between ZYC-ZFe and ZYC-Zn. Symbol / indicates that relevant data were not detected.

**Table 5:** Fine analysis of the pore structure based on the NLDFT and QSDFT models.

Parameters	Unit	ZYC-ZFe	ZYC-Zn
NLDFT micropore distribution			
<0.7 nm	cm^3^/g	0.182 ± 0.004	0.175 ± 0.003
0.7–1.0 nm	cm^3^/g	0.203 ± 0.004	0.210 ± 0.004
1.0–2.0 nm	cm^3^/g	0.080 ± 0.002	0.072 ± 0.001
QSDFT mesoporous distribution			
2–5 nm	cm^3^/g	0.285 ± 0.005	0.180 ± 0.003
5–10 nm	cm^3^/g	0.247 ± 0.005	0.165 ± 0.003
10–50 nm	cm^3^/g	0.126 ± 0.003	0.112 ± 0.002
Surface energy heterogeneity			
Adsorption potential distribution width (ε distribution)	kJ/mol	8.2 ± 0.2	6.5 ± 0.2
Connectivity index	-	0.78 ± 0.02	0.65 ± 0.02

**Table 6:** Langmuir and Dubinin–Astakhov (D–A) model fitting parameters for methanol adsorption.

Carbons	*Q*_L_*	*R* ^2^	*Q*_D−A_^*^	*E*	*n*	*R* ^2^
	mg/g		mg/g	kJ/mol		
ZYC-ZFe	713.06 ± 15.23	0.9991	744.11 ± 1.55	17.55 ± 0.17	2.20 ± 0.04	0.9988
ZYC-Zn	613.78 ± 18.57	0.9928	631.45 ± 3.02	14.19 ± 0.11	1.95 ± 0.05	0.9985
IM-WNC [19]	688.99 ± 13.52	0.9925	/	/	/	/
PM-WNC [19]	625.98 ± 11.44	0.9964	/	/	/	/
WNC-4 [28]	552.67 ± 23.83	0.9939	/	/	/	/
GHSC [27]	534.94 ± 6.411	0.9915	/	/	/	/

**Table 7:** Adsorption kinetics parameters calculated by the Sokoda–Suzuki model.

Carbons	Adsorption			Desorption		
	*T* (°C)	Q* (mg/g)	*D*_S0_ (10^−10^ m^2^/s)	*T* (°C)	Q* (mg/g)	*D*_S0_ (10^−10^ m^2^/s)
ZYC-ZFe	15	580.61 ± 4.38	1.4 × 10^−10^	75	584.18 ± 3.96	1.52
	20	552.34 ± 4.12	1.6 × 10^−10^	85	596.03 ± 7.24	1.76
	25	526.47 ± 3.89	1.8 × 10^−10^	90	610.53 ± 5.97	1.98
ZYC-Zn	15	374.87 ± 2.21	0.87 × 10^−10^	75	490.57 ± 5.18	0.98
	20	356.21 ± 2.08	0.98 × 10^−10^	85	501.73 ± 6.45	1.15
	25	338.95 ± 1.96	1.1 × 10^−10^	90	511.18 ± 3.96	1.32

**Table 8:** Thermodynamic parameters and activation energies for methanol adsorption–desorption.

Carbons	Adsorption				Desorption					
	*T*	Δ*G*_ads_	Δ*H*_ads_	Δ*S*_ads_	*T*	Δ*G*_des_	Δ*H*_des_	Δ*S*_des_	*E*_a_	*E*_d_
	K	kJ/mol	kJ/mol	J/(mol·K)^−1^	K	kJ/mol	kJ/mol	J/(mol·K)^−1^	kJ/mol	kJ/mol
ZYC-ZFe	288.15	−9.90	−38.72	−99.9	348.15	1.39	−38.72	104.8	10.2	12.8
	293.15	−8.71		−102.4	358.15	1.01		104.8		
	298.15	−7.46		−104.8	363.15	0.67		104.8		
ZYC-Zn	288.15	−7.23	−32.15	−86.4	348.15	0.90	−38.72	90.4	9.5	12.1
	293.15	−6.41		−87.8	358.15	0.60		90.4		
	298.15	−5.20		−90.4	363.15	0.33		90.4		

**Table 9:** Core performance comparison of 10 adsorbents under a 30 min cycle.

Sample	Saturated Adsorption Capacity *Q*_L_* (mg/g)	Actual Desorption Capacity (mg/g)	Effective Contribution Ratio *η* (%)	Unit Cooling Capacity *Q*_ref_ (kJ/kg)	Specific Cooling Power *N*_ref_ (kJ/(kg·h))	*COP*
ZYC-ZFe	713.06 ± 15.23	636.05 ± 12.85	89.20	812.4 ± 12.5	1534.6 ± 24.3	0.52
ZYC-Zn	613.78 ± 18.57	469.54 ± 10.23	76.50	590.7 ± 9.8	1025.8 ± 17.2	0.38
IM-WNC [19]	688.99 ± 13.52	557.94 ± 11.02	80.98	501.43 ± 10.03	799.06 ± 15.98	0.35
PM-WNC [19]	625.98 ± 11.44	471.69 ± 8.75	75.35	433.70 ± 8.67	673.22 ± 13.46	0.30
WNC-4 [28]	552.67 ± 23.83	308.45 ± 8.84	55.81	281.75 ± 8.07	563.50 ± 16.15	0.27
GHSC [27]	482.85 ± 3.85	299.37 ± 5.21	62.00	441.05 ± 3.53	882.07 ± 8.27	0.41

**Table 10:** *COP* comparison of 10 adsorbents at different desorption temperatures.

Sample	60 °C	70 °C	80 °C	90 °C	100 °C	120 °C
ZYC-ZFe	0.35	0.42	0.48	0.52	0.55	0.61
ZYC-Zn	0.21	0.28	0.34	0.38	0.42	0.48
IM-WNC [19]	0.18	0.25	0.31	0.35	0.39	0.46
PM-WNC [19]	0.12	0.19	0.25	0.30	0.34	0.41
WNC-4 [28]	0.03	0.09	0.18	0.27	0.35	0.44
GHSC [27]	0.06	0.13	0.24	0.41	0.49	0.60

## Data Availability

The original contributions presented in this study are included in the article/Supplementary Materials. Further inquiries can be directed to the corresponding authors.

## Supplementary Materials

The following supporting information can be downloaded at: https://www.mdpi.com/article/10.3390/ma19153281/s1, Figure S1: Temperature contour of adsorbent beds with diameters of 100 mm, 200 mm, and 400 mm at a heating water temperature of 370 K. Table S1: Summary of D-optimal experimental design and results for the carbonization stage; Table S2: Analysis of variance for the quadratic model for the carbonization stage; Table S3: Results of response surface methodology experiments for the activation stage; Table S4: Analysis of variance response surface methodology experiments for the activation stage; Table S5: Fourier transform infrared spectroscopy (FTIR) peak assignments and analysis; Table S6: Fitting parameters for high-resolution XPS peak deconvolution; and Table S7: Quantitative analysis of surface functional groups (Boehm titration method).

## Abbreviations

The following abbreviations are used in this manuscript:

TCM	Traditional Chinese medicine
TCM residues	Traditional Chinese medicine residues
*S*_BET_	BET specific surface area
*V*_T_	Total pore volume
*COP*	Coefficient of performance
RSM	Response surface methodology
AM	Astragalus membranaceus
GU	Glycyrrhiza uralensis
PL	Pueraria lobata
AS	Angelica sinensis
ZYC-ZFe	In situ Fe-doped hierarchically porous carbon derived from Astragalus and licorice residues
ZYC-Zn	ZnCl_2_ single impregnation control carbon material
BBD	Box–Behnken design
DOD/D-optimal	D-optimal design
ANOVA	Analysis of variance
*α*	Impregnation ratio (carbonization stage)
*C*	Impregnation concentration (g/L)
*T*_c_	Carbonization temperature (°C)
*T*/*t*	Temperature rise rate (°C/min)
*t*	Carbonization holding time (min)
*γ*	Burn-off rate (%)
*β*	Phosphoric acid solution impregnation ratio (mL/kg)
*T*_h_	Activation temperature (°C)
*t*_h_	Activation holding time (min)
SEM	Scanning electron microscopy
XPS	X-ray photoelectron spectroscopy
FTIR	Fourier transform infrared spectroscopy
*Q*_ad_	Methanol adsorption capacity (mg/g)
*Q*_de_	Methanol desorption capacity (mg/g)
*Q*	Methanol adsorption/desorption amount at time t (mg/g)
*Q**	Equilibrium adsorption/desorption amount (mg/g)
*k*	Adsorption rate constant (s^−1^)
*D*_S0_	Surface diffusion coefficient (m^2^/s)
*E*_a_	Activation energy for surface diffusion (J/mol)
*R*_p_	Average particle diameter of carbon material (m)
*T*	Absolute temperature (K)
*Q*_L_	Methanol adsorption/desorption amount (Langmuir model, mg/g)
*k*_L_	Langmuir adsorption constant
*Q*_L_^*^	Maximum monolayer adsorption capacity (mg/g)
*p*	Actual gas pressure (Pa)
*p*_0_	Saturation vapor pressure of methanol at the test temperature (Pa)
*Q*_D-A_	Methanol adsorption/desorption amount (D–A model, mg/g)
*Q*_D-A_^*^	Maximum adsorption capacity (D–A model, mg/g)
*ε*	Adsorption potential
*E*	Characteristic adsorption energy (J/mol)
*n*	Empirical parameter of the D-A model
*Q*_ref_	Cooling capacity per unit mass of adsorbent (kJ/kg)
*V*	Desorbed refrigerant volume per kilogram of carbon material (L/kg)
Δ*H*_ev_	Latent heat of vaporization of methanol (J/mL)
*W*_ad_	Mass of carbon adsorbent (kg)
*N*_ref_	Specific cooling power (kJ/(kg·h))
*t*_ad_	Total duration of a single adsorption/desorption cycle (min)
TLC	Total lignocellulose content
*S*_mi_	Micropore specific surface area (m^2^/g)
*S*_ex_	External specific surface area (m^2^/g)
*V*_mi_	Micropore volume (cm^3^/g)
*V*_mes_	Mesopore volume (cm^3^/g)
*R*_mes_	Mesopore ratio (%)
*D*	Average pore diameter (Å)
*h*_f_	Ash content (%)
*λ*	Thermal conductivity (W/(m·K))
NLDFT	Non-local density functional theory
QSDFT	Quenched solid density functional theory
*p*H_pzc_	Point of zero charge
Δ*H*	Adsorption enthalpy (kJ/mol)
Δ*G*	Gibbs free energy change (kJ/mol)
Δ*S*	Entropy change (kJ/(mol·K))
*η*	Effective contribution ratio
IM-WNC	In situ Fe-doped municipal biological sludge carbon
PM-WNC	Post-impregnated Fe-doped municipal biological sludge carbon
WNC-4	Ordinary municipal sludge carbon
GHSC	Graphene-doped coal-based carbon
BET	Brunauer–Emmett–Teller model
BJH	Barrett–Joyner–Halenda model
S–S model	Sokoda–Suzuki kinetic model
D–A model	Dubinin–Astakhov adsorption isotherm model
STP	Standard temperature and pressure
