# Supplementary material for: In Situ Fe-Doped Hierarchically Porous Carbon Derived from Astragalus and Licorice Residues: Pore Engineering and Thermal Conductivity Enhancement for Methanol Adsorption Refrigeration
Source: Materials (Basel). 2026 Aug 3;19(15):3281. doi: 10.3390/ma19153281 (PMC13467731; doi:10.3390/ma19153281)
Supplement: Supplementary file 1 [file materials-19-03281-s001.zip › materials-4430341-supplementary.pdf]

Supplementary Materials:

Table S1. Summary of D-optimal experimental design and results for the carbonization stage.

Table S2. Analysis of variance for the quadratic model for the carbonization stage..

Table S3. Results of response surface methodology experiments for the activation stage.

Table S4. Analysis of variance response surface methodology experiments for the activation stage.

Table S5. Fourier transform infrared spectroscopy (FTIR) peak assignments and analysis.

Table S6. Fitting parameters for high-resolution XPS peak deconvolution.

Table S7. Quantitative analysis of surface functional groups (Boehm titration method).

Figure S1. Temperature contour of adsorbent beds with diameters of 100 mm, 200 mm, and 400 mm at a heating water temperature of 370 K.

Table S1. Summary of D-optimal experimental design and results for the carbonization stage.

No.	Factors and Levels					Response to target		
	α	C	Tc	T/t	t	V	S_{BET}	γ
	/	g/L	℃	℃/min	min	cm^3/g	m^2/g	%
1	3.0	50	400	20	80	0.288	467	35.22
2	1.0	50	400	20	60	0.406	527	45.16
3	2.0	100	400	15	80	0.388	488	44.23
4	2.0	50	400	15	120	0.306	503	54.26
5	1.0	100	500	15	60	0.588	610	61.25
6	1.0	75	400	10	120	0.404	527	43.25
7	2.0	50	500	20	60	0.466	524	53.17
8	2.0	50	600	10	60	0.577	584	55.62
9	2.0	75	600	10	80	0.622	701	55.23
10	1.0	50	600	15	80	0.547	589	51.25
11	3.0	50	500	15	120	0.367	560	47.89
12	3.0	50	400	15	60	0.298	467	45.03
13	1.0	75	500	20	60	0.596	632	50.45
14	1.0	50	600	10	120	0.556	621	55.12
15	3.0	100	400	20	60	0.208	470	50.25
16	2.0	100	600	15	60	0.433	568	53.16
17	2.0	50	400	10	80	0.478	554	45.25
18	1.0	75	500	15	120	0.617	652	57.02
19	2.0	75	600	15	120	0.588	638	55.03
20	3.0	100	500	15	80	0.297	420	61.25
21	1.0	75	500	10	80	0.608	634	55.02
22	1.0	75	600	20	80	0.632	696	57.25
23	3.0	100	500	10	120	0.286	540	60.21
24	3.0	75	500	15	60	0.427	610	47.65
25	1.0	50	500	10	60	0.558	621	50.17
26	2.0	50	500	10	120	0.533	600	52.23
27	2.0	100	500	10	80	0.396	523	50.25
28	3.0	75	500	20	120	0.447	628	49.16
29	1.0	100	600	10	80	0.567	602	63.17
30	3.0	50	500	10	80	0.332	533	47.25
31	1.0	50	500	20	80	0.517	602	49.25
32	1.0	100	500	20	120	0.61	634	60.17
33	1.0	100	600	20	60	0.527	617	60.25
34	1.0	100	400	10	60	0.406	580	50.25
35	2.0	75	400	15	60	0.481	567	47.16
36	1.0	75	600	15	60	0.61	667	53.24
37	2.0	100	600	20	80	0.395	506	45.69
38	1.0	100	400	20	80	0.441	596	50.88
39	1.0	75	400	15	80	0.525	633	50.02
40	1.0	100	600	15	120	0.458	594	64.14
41	3.0	50	600	20	60	0.417	582	47.23
42	2.0	50	600	20	120	0.561	574	58.63
43	2.0	50	500	15	80	0.545	574	53.26
44	3.0	100	600	20	120	0.153	450	68.25

45	2.0	100	500	15	120	0.375	517	50.22
46	3.0	50	400	10	120	0.311	524	51.03
47	3.0	100	600	10	60	0.302	580	57.16
48	3.0	75	600	10	120	0.442	637	51.26
49	3.0	75	400	10	80	0.396	587	44.25
50	2.0	75	400	20	120	0.502	589	48.56
51	2.0	75	500	20	80	0.596	617	52.15
52	2.0	100	400	10	120	0.406	495	45.16
53	2.0	75	500	10	60	0.433	582	57.16
54	3.0	100	400	15	120	0.28	500	62.05
55	3.0	75	600	15	80	0.432	607	50.25
56	2.0	75	600	20	60	0.543	647	52.15

Table S2. Analysis of variance for the quadratic model for the carbonization stage.

Analysis of variance for the quadratic model of V_T					
Source	Sum of Squares	*df*	Mean Square	*F*-value	*p*-value
Model	0.6012	6	0.1002	32.37	< 0.0001
A-α	0.3371	2	0.1686	54.44	< 0.0001
B-*C*	0.1235	2	0.0617	19.94	< 0.0001
C-*T*c	0.0847	2	0.0424	13.68	< 0.0001
Residual	0.1517	49	0.0031		
Cor Total	0.7529	55			
Analysis of variance for the quadratic model of S_{BET}					
Source	Sum of Squares	*df*	Mean Square	*F*-value	*p*-value
Model	1.502E+05	6	25028.31	19.73	< 0.0001
A-α	38773.18	2	19386.59	15.29	< 0.0001
B-*C*	62981.10	2	31490.55	24.83	< 0.0001
C-*T*c	32902.49	2	16451.24	12.97	< 0.0001
Residual	62144.96	49	1268.26		
Cor Total	2.123E+05	55			
Analysis of variance for the quadratic model of γ					
Source	Sum of Squares	*df*	Mean Square	*F*-value	*p*-value
Model	1580.28	10	158.03	14.15	< 0.0001
A-α	45.83	2	22.92	2.05	0.1404
B-*C*	315.36	2	157.68	14.11	< 0.0001
C-*T*c	487.14	2	243.57	21.80	< 0.0001
AB	643.40	4	160.85	14.40	< 0.0001
Residual	502.74	45	11.17		
Cor Total	2083.02	55			

Table S3. Results of response surface methodology experiments for the activation stage.

Run	**Factors and Levels**			**Response to target**	
	β	T_h	t_h	V	S_{BET}
	mL/kg	°C	min	cm³/g	m²/g
1	650	875	100	1.123	1244.69
2	650	950	80	0.914	1057.18
3	650	875	100	1.056	1197.88
4	500	875	80	0.886	974.56
5	800	800	100	0.881	946.23
6	650	950	120	0.902	1056.74
7	500	875	120	0.88	923.57
8	650	875	100	1.12	1256.71
9	800	875	120	0.881	947.69
10	500	950	100	0.886	967.13
11	650	875	100	1.142	1233.17
12	650	800	120	1.006	1156.47

Run	Factors and Levels			Response to target	
	β	T_h	t_h	V	S_{BET}
	mL/kg	°C	min	cm³/g	m²/g
13	800	875	80	0.843	957.82
14	650	800	80	1.104	1187.62
15	650	875	100	1.087	1204.56
16	500	800	100	1.017	1114.14
17	800	950	100	0.847	1024.63

Table S4. Analysis of variance response surface methodology experiments for the activation stage.

Analysis of variance for the quadratic model of S_{BET}					
Source	Sum of Squares	df	Mean Square	F-value	*p*-value
Model	2.244E+05	9	24936.07	22.70	0.0002
A-β	1326.90	1	1326.90	1.21	0.3081
B-T_h	11158.69	1	11158.69	10.16	0.0153
C-t_h	1074.39	1	1074.39	0.9779	0.3557
AB	12702.42	1	12702.42	11.56	0.0114
AC	417.38	1	417.38	0.3799	0.5572
BC	235.78	1	235.78	0.2146	0.6572
A^2	1.504E+05	1	1.504E+05	136.87	< 0.0001
B^2	2714.00	1	2714.00	2.47	0.1600
C^2	32244.95	1	32244.95	29.35	0.0010
Residual	7690.43	7	1098.63		
Lack of Fit	5106.02	3	1702.01	2.63	0.1863
Pure Error	2584.41	4	646.10		
Cor Total	2.321E+05	16			
Analysis of variance for the quadratic model of *V*					
Source	Sum of Squares	df	Mean Square	F-value	*p*-value
Model	0.1832	9	0.0204	12.44	0.0016
A-β	0.0059	1	0.0059	3.60	0.0997
B-T_h	0.0263	1	0.0263	16.10	0.0051
C-t_h	0.0008	1	0.0008	0.4648	0.5173
AB	0.0024	1	0.0024	1.44	0.2695
AC	0.0005	1	0.0005	0.2958	0.6034
BC	0.0018	1	0.0018	1.13	0.3230
A^2	0.0991	1	0.0991	60.58	0.0001
B^2	0.0083	1	0.0083	5.08	0.0589
C^2	0.0267	1	0.0267	16.34	0.0049
Residual	0.0115	7	0.0016		
Lack of Fit	0.0068	3	0.0023	1.96	0.2625
Pure Error	0.0046	4	0.0012		
Cor Total	0.1947	16			

Table S5. Fourier transform infrared spectroscopy (FTIR) peak assignments and analysis.

Wave number	Family Affiliation	Relative peak intensity of

(cm^{-1})		ZYC-ZFe (based on ZYC-Zn)
3420~3450	O-H stretching vibration (hydroxyl, carboxyl)	1.52
2920/2850	Saturation C-H stretching vibration (methylene, methyl)	1.21
1700~1720	C=O stretching vibration (carboxyl group, carbonyl group)	1.68
1610~1630	The stretching vibration of the aromatic ring C=C	1.34
1380~1400	O-H bending vibration (phenolic hydroxyl group)	1.47
1160~1200	C-O stretching vibration (ether, ester, alcohol)	1.59
1030~1050	P-O/P=O stretching vibration (phosphate-activated residue)	1.07
570~590	Fe-O stretching vibration (characteristic peak of Fe_3O_4)	Only ZYC-ZFe was detected

Table S6. Fitting parameters for high-resolution XPS peak deconvolution.

Peak splitting	Carbon	Peak assignment	Peak position (eV)	half-width (eV)	Peak Area Ratio (%)
C1s (280~292 eV)	ZYC-ZFe	C-C/C=C	284.8	1.22	61.2
		C-O	286.2	1.35	22.7
		C=O	287.6	1.41	10.5
		O-C=O	288.9	1.38	5.6
	ZYC-Zn	C-C/C=C	284.8	1.18	67.5
		C-O	286.1	1.32	18.3
		C=O	287.5	1.37	9.2
		O-C=O	288.8	1.35	5.0
O1s (528~536 eV)	ZYC-ZFe	C=O	531.2	1.31	28.4
		-OH	532.3	1.42	47.2
		C-O-C/O-C=O	533.5	1.37	24.4
	ZYC-Zn	C=O	531.1	1.28	25.7
		-OH	532.2	1.39	43.8
		C-O-C/O-C=O	533.4	1.35	30.5
Fe 2p high-resolution spectrum (705-735eV)	ZYC-ZFe	Fe^{2+} 2p3/2	710.2	3.2	20. 3
		Fe^{3+} 2p3/2	711.8	3.5	44. 0
		Satellite Peak（2p3/2)	715.5	3.8	2. 9
		Fe^{2+} 2p1/2	723.5	3.3	7. 1
		Fe^{3+} 2p1/2	725.2	3.6	23. 5
		Satellite Peak（2p1/2)	728.8	3.7	2. 3
Zn2p (1018-1050 eV)	ZYC-ZFe	Zn^{2+} 2p3/2	1021.6	2.10	66. 7
		Zn^{2+} 2p1/2	1044.7	2.30	33. 3
	ZYC-Zn	Zn^{2+} 2p3/2	1021.5	2.00	62.8
		Zn^{2+} 2p1/2	1044.6	2.20	31.4
		Satellite (Zn 2p)	1047.3	3.50	5.8

Table S7. Quantitative analysis of surface functional groups (Boehm titration method).

**Functional Group Type**	**ZYC-ZFe**	**ZYC-Zn**
	**mmol/g**	**mmol/g**

Carboxyl group (-COOH)	0.72 ± 0.05	0.49 ± 0.04
Lactone group (-COO-)	0.51 ± 0.04	0.39 ± 0.03
phenol hydroxyl group OH	0.95 ± 0.06	0.76 ± 0.05
Carbonyl (C=O)	0.25 ± 0.03	0.18 ± 0.02
Total acidic groups	2.43 ± 0.10	1.82 ± 0.11
Total basic groups	0.38 ± 0.03	0.45 ± 0.03
Point of zero electric charge (pH_{pzc})	5.8 ± 0.2	6.5 ± 0.2

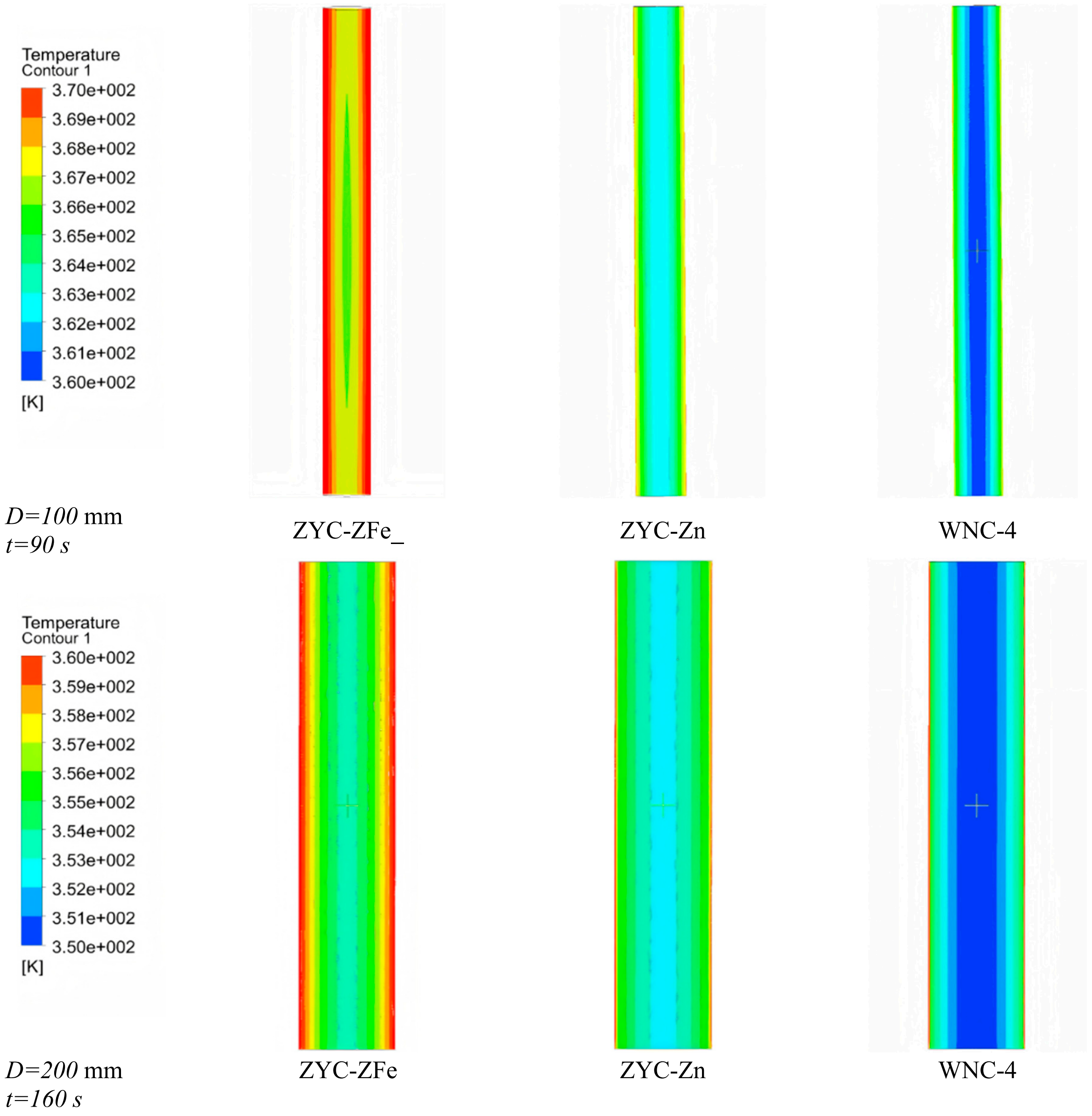

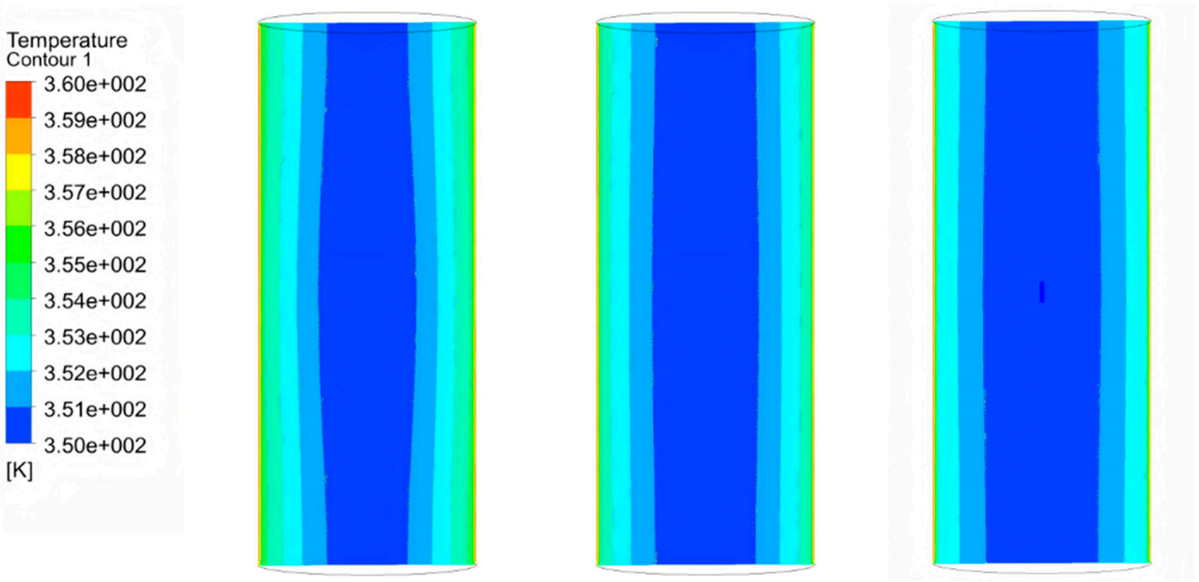

D=400 mm
t=300 s

ZYC-ZFe ZYC-Zn WNC-4

Figure S1. Temperature contour of adsorbent beds with diameters of 100 mm, 200 mm, and 400 mm at a heating water temperature of 370 K.
